# Autophagic sequestration of SQSTM1 disrupts the aggresome formation of ubiquitinated proteins during proteasome inhibition

**DOI:** 10.1038/s41419-022-05061-8

**Published:** 2022-07-15

**Authors:** Chenliang Zhang, Chen Huang, Hongwei Xia, Huanji Xu, Qiulin Tang, Feng Bi

**Affiliations:** 1 Laboratory of Molecular Targeted Therapy in Oncology, West China Hospital of Sichuan University, Chengdu, China; 2 Department of Medical Oncology, Cancer Center, West China Hospital of Sichuan University, Chengdu, China

**Keywords:** Cell biology, Cancer therapy, Proteasome

## Abstract

Aggresome formation is a protective cellular response to counteract proteasome dysfunction by sequestering misfolded proteins and reducing proteotoxic stress. Autophagic degradation of the protein aggregates is considered to be a key compensating mechanism for balancing proteostasis. However, the precise role of autophagy in proteasome inhibition-induced aggresome biogenesis remains unclear. Herein, we demonstrate that in the early stage of proteasome inhibition, the maturation of the autophagosome is suppressed, which facilitates aggresome formation of misfolded proteins. Proteasome inhibition-induced phosphorylation of SQSTM1 T269/S272 inhibits its autophagic receptor activity and promotes aggresome formation of misfolded proteins. Inhibiting SQSTM1 T269/S272 phosphorylation using Doramapimod aggravates proteasome inhibitor-mediated cell damage and tumor suppression. Taken together, our data reveal a negative effect of autophagy on aggresome biogenesis and cell damage upon proteasome inhibition. Our study suggests a novel therapeutic intervention for proteasome inhibitor-mediated tumor treatment.

## Introduction

The clearance of misfolded proteins is critical for cells to maintain proteostasis. The ubiquitin–proteasome system (UPS) is the major pathway responsible for degrading misfolded proteins following polyubiquitination [1, 2]. Many types of cellular stress, such as ER stress, oxidative stress, and proteasomal inhibition, can induce the over-synthesis of misfolded proteins as well as proteasome activity insufficiency [3–5]. However, cells can counteract this proteotoxic stress through the aggregation and autophagic degradation of misfolded proteins [6]. Proteasome inhibition can not only induce the aggregation of polyubiquitinated proteins but also further induce the transfer of protein aggregates to the microtubule organizing center (MTOC) to form perinuclear aggresomes [7, 8]. This centralization process is considered to be a protective mechanism for cells in response to proteasome impairment [7, 9, 10].

Macroautophagy (hereafter autophagy) has been formerly considered a protective mechanism to maintain proteostasis under these stresses that induce protein aggregation [6, 11]. However, recent studies have revealed that during proteasome inhibition, the activation of autophagy occurred after aggresome formation of misfolded proteins and that cell survival did not benefit from activated autophagy but instead depended on the formation of aggresome [12]. This evidence suggests an unconcerted action of autophagy in its activation and misfolded protein degradation upon proteasome inhibition. It is unclear whether autophagy affects protein aggregation or aggresome formation. The accumulation of misfolded protein aggregates is the dominant contributing factor to neuron damage in several neurodegenerative diseases, such as Parkinson’s and Huntington’s [13]. Moreover, proteasome inhibitors have been developed for cancer treatment based on the importance of proteasomal activity in tumor growth [14–16]. Besides, aggresome formation of misfolded proteins has been reported to mediate drug resistance in proteasome inhibitor-treated cancer cells [17, 18]. Therefore, understanding the crosstalk mechanism between autophagy and proteostasis regulation is beneficial for understanding the pathogenesis of cell death and overcoming drug resistance.

Numerous studies in cultured cells have revealed that there are two main steps involved in aggresome formation following proteasome impairment. First, proteasome inhibition induces the packaging of misfolded proteins (often polyubiquitinated) into micro-aggregates, in which E3 ubiquitin ligase, such as CHIP, molecular chaperones, and their regulating co-chaperones, such as HSP70, HSPB8, and BAG3, play a key role [19–21]. Second, the micro-aggregates are transported along the microtubule network to the microtubule organizing center (MTOC) to form an aggresome [22, 23]. Although the mechanism of micro-aggregate transport is not well understood, HDAC6 is considered a critical regulatory factor in this process. HDAC6 can bind to both polyubiquitinated misfolded proteins and dynein motors using its BUZ domain and DMB domain, respectively, thereby recruiting misfolded proteins to dynein motors for transport [8, 24, 25].

SQSTM1, a multifunctional scaffold protein, plays an important role in activating diverse signaling pathways, such as oxidative stress, selective autophagy, and nutritional metabolism [26, 27]. Several functional domains present in SQSTM1, such as the Phox and Bem1 (PB1) domains, ubiquitin association (UBA) domain, and microtubule-associated protein light chain 3 (LC3)-interacting region (LIR) domain, could mediate the protein-protein interaction. As an autophagy receptor, SQSTM1 binds to polyubiquitinated cargos and presents them to the autophagosome by interacting with LC3/Atg8, thereby promoting the degradation of selective substrates [28]. In this process, multiple kinases have been reported to affect SQSTM1 functions by affecting its phosphorylation level at different amino acid sites. The phosphorylation of SQSTM1 at Ser28 by Pink1-s (short form of PTEN-induced putative kinase 1) and Ser403 by both CK2 (casein kinase 2) and TBK1 (TANK-binding kinase 1) simulates the binding activity of SQSTM1 to ubiquitinated cargos [29–32]. In addition, autophagy kinase ULK1 (unc-51-like autophagy-activating kinase 1) reportedly phosphorylates SQSTM1 at a novel site Ser407 (equivalent to Ser409 in mice) to destabilize the dimer of the UBA domain and enhance the affinity of SQSTM1 to ubiquitin [33]. Our previous studies demonstrated that p38δ-mediated SQSTM1 phosphorylation at T269/S272 sites promoted the aggresome formation of ubiquitinated protein aggregates following proteasome inhibition [34].

This study investigated the relationship between the aggresome formation of polyubiquitinated proteins and autophagy following proteasome inhibition. We showed that autophagy could disrupt the aggresome formation induced by proteasome inhibitors. Phosphorylation of SQSTM1 T269/S272 could promote protein aggregates transport to the aggresome by blocking their presentation in autophagosomes. Taken together, our results suggest that autophagy might negatively affect the protective mechanism of cells against proteasome inhibition.

## Materials and methods

### Cell culture and transfection

Wild-type AD293, ATG5 knockout (ATG5^−/−^) AD293, SQSTM1 knockout (SQSTM^−/−^) AD293 and SQSTM^−/−^ re-expressing cell lines were obtained and constructed as described in our previous study [29, 34]. HeLa, MDA-MB-231, HCT-116, and A375 cells were purchased from ATCC. HEK293FT cell line was obtained from Thermo Fisher Scientific and used for lentivirus production. All cells were cultured at 37 °C in Dulbecco’s modified Eagle’s medium (DMEM, Gibco, 12800082) supplemented with 10% fetal bovine serum (FBS, HyClone, SH30071.03), 1% penicillin–streptomycin (Thermo Fisher Scientific, 15140211), 2 mM l-glutamine (Thermo Fisher Scientific, 25030081), and 1× non-essential amino acids (NEAAs, Thermo Fisher Scientific, 11140076) in a 5% CO_2_ incubator. All the cell lines were authenticated and tested for contamination.

Plasmid transfection was carried out with Megatran (OriGene, TT200003) for AD293 and HeLa cells, while Lipofectamine 2000 (Thermo Fisher Scientific, 11668019) was utilized for A375 cells. All siRNA transfections were carried out with Lipofectamine 2000. All transfections were performed according to the manufacturer’s instructions.

### Plasmids

pcDNA3.1-FLAG-SQSTM1 (WT, T269A/S272A, T269E/S272D), pcDNA3.1-mRFP-CFTR∆F508, pcDNA3.1-FLAG-p38δ (WT, K54R, F324S) and pcDNA3.1-Myc-p38δ (WT, K54R, F324S) were constructed as previously described [29, 34]. To generate mutant Huntingtin expression constructs, a fragment of huntingtin exon 1 with 98 polyglutamine repeats (HttQ98) fused EGFP were cloned into pcDNA3.1 vector. Other mammalian expression vectors were generated by inserting ORF Fragments amplified by PCR from the genes of interest into the expression vector pcDNA3.1 with FLAG or EGFP tag. All the plasmids were verified by DNA sequencing. Primer sequence information used for ORF amplification is listed in Supplementary Table S1.

### Chemical reagents

The following reagents were used: MG132 (Merck Millipore, 474790); Bafilomycin A1 (CSNpharm, CSN10374); SBI-0206965 (CSNpharm, CSN16884); Bortezomib (CSNpharm, CSN10115); Doramapimod (CSNpharm, CSN10856); Wortmannin (MCE, HY-10197); SB203580 (MCE, HY-10256); SB202190 (HY-10295); Rapamycin (MCE, HY-10219); Torin 2 (MCE, HY-13002); Asciminib (Topscience, T5177); BRAF inhibitor (MCE, HY-10247); Chloroquine (MCE, HY-17589A).

### ATG5 knockdown

The siRNA targeting ATG5 (sequences as flowing: 5′-GACGUUGGUAACUGACAAA-3′) and the scrambled siRNA (siControl) were synthesized by Gima Company. Cells were transfected with 50 nM siRNA using lipofectamine 2000. After 48 h, the transfected cells were used for experiments. The knockdown efficiency was analyzed by western blot.

### Protein extraction, immunoprecipitation, and western blot analysis

The total protein extract was prepared by homogenizing the cells in a 1× SDS sample buffer. The NP-40-soluble and -insoluble protein fractions from the cells were prepared as previously described [34]. Immunoprecipitation and western blot were carried out as previously described [34]. Primary antibodies against the following proteins were used for western blot analysis: K48-linked Ub chain-specific antibody (Millipore, 05-1307); K63-linked Ub chain-specific antibody (Abcam, ab179434); ubiquitin (Abcam, ab134953); Phospho-SQSTM1 (Thr269/Ser272)-specific antibody (Phosphosolutions, P196-269); SQSTM1 (Santa Cruz Biotechnology (sc-28359); ATG5 (Cell Signaling Technology, 12994); LC3B (Cell Signaling Technology, 3868); ATG16L1 (Abcam, ab187671); WIPI2 (Abcam, ab105459); Beclin1 (Proteintech, 11306-1-AP); GFP (Rockland, 600-101-215); FLAG tag (Prospec, ANT-146-b); Myc Tag (Biolegend, MMS-150R); GAPDH (Zen Bioscience, 200306); β-actin (Zen Bioscience, 200068-6D7). See Supplementary Table S2 for further details and dilutions of all antibodies.

### Immunostaining

Cells were grown onto sterile coverslips placed in 24- or 48-well plates. After treatment, cells were fixed for 15 min in 4% paraformaldehyde, permeabilized for 15 min with 0.2% Triton X-100 (Merck Millipore, 9410-1 L), and then blocked with 5% goat serum (Jackson ImmunoResearch Laboratories, 005-000-12) for 1 h. Cells were incubated with primary antibodies for 2 h at room temperature or overnight at 4 °C followed by incubation for 1 h with appropriate secondary antibodies. Their nuclei were then stained with DAPI solution, and they were mounted on slides for fluorescence microscopy. Images were captured with a fluorescent microscope (Nikon Eclipse 80i equipped with Nikon PLAN FLUOR ×40 objective) or Nikon confocal microscope (Nikon, N-STORM & A1). Photographic images were resized and analyzed by ImageJ software. The following primary antibodies were used for immunostaining: SQSTM1 (Santa Cruz Biotechnology, sc-28359); K48-linked Ub chain-specific antibody (Merck Millipore, 05-1307); ATG5 (Cell Signaling Technology, 12994), WIPI2 (Abcam, ab105459); γ-tubulin (Novus, NBP2-43585); FLAG Tag (Propec, ant-146-b). See Supplementary Table S2 for further details and dilutions of all antibodies.

### Transmission electronic microscopy

After treatment, the cells were prefixed with a 3% glutaraldehyde, then postfixed in 1% osmium tetroxide, dehydrated in series acetone, infiltrated in Epox 812 for a longer, and embedded. The semithin sections were stained with methylene blue and Ultrathin sections were cut with a diamond knife, and stained with uranyl acetate and lead citrate. Sections were examined with JEM-1400-FLASH Transmission Electron Microscope.

### Lentivirus generation and infection

To generate stable gene expression cell lines, the DNA fragments corresponding to the ORFs of expressing genes were cloned into pLVX-puro lentiviral vectors. Lentiviral packaging and stable cell selection were carried out as described previously [34].

### Cell viability assay

Cells were seeded into 96-well plates at a density of 3 × 10^3^/well. After 24 h, the cells were subsequently treated with the indicated reagent. Cell viability was determined by Cell Counting-8 Kit (Dojindo, Kumamoto, Japan) as recommended by the manufacturers.

### Colony formation assay

Cells were seeded in 12-well plates at a density of 1 × 10^3^/well. The cells were allowed to grow for 2 days and then incubated with various treatments for 1 week to allow colony formation. Cells were fixed with methanol for 20 min and stained with 0.1% crystal violet for 30 min. After being washed with PBS, the picture of the plates was taken, and the colony area was quantified using ImageJ.

### Tumor xenograft analysis

All animal experiments complied with ethical regulations and were approved by the subcommittee on research animal care of Western China Hospital of Sichuan University. Nude mice (nu/nu, 5-week-old females) were injected subcutaneously with 5 × 10^6^ A375 cells. The tumors were allowed to grow for 10 days, and drug treatment was then administered accordingly. The mice were randomly divided into four groups: control (saline), Bortezomib (0.4 mg/kg, intraperitoneally, twice/week), Doramapimod (10 mg/kg, orally, twice/week), and a combination of Bortezomib (0.4 mg/kg, intraperitoneally, twice/week) and Doramapimod (10 mg/kg, orally, twice/week). The tumor was measured twice weekly using digital calipers [tumor volume = 1/2 (length × width^2^)]. Mice were killed on day 38, and the tumors were dissected and analyzed.

### Statistical analyses

Data were represented as mean ± SEM. Comparisons between individual data points were made using a two-tailed Student *t* test (2 groups) or one-way ANOVA analysis (>2 groups) with posthoc (Dunnett’s multiple comparisons test) analysis. Differences were considered statistically significant when *P* < 0.05. All statistical analyses were performed using Graph Prim 7.0.

## Results

### Suppressing autophagy is the early response of cells to proteasome inhibition

**Fig. 1:**
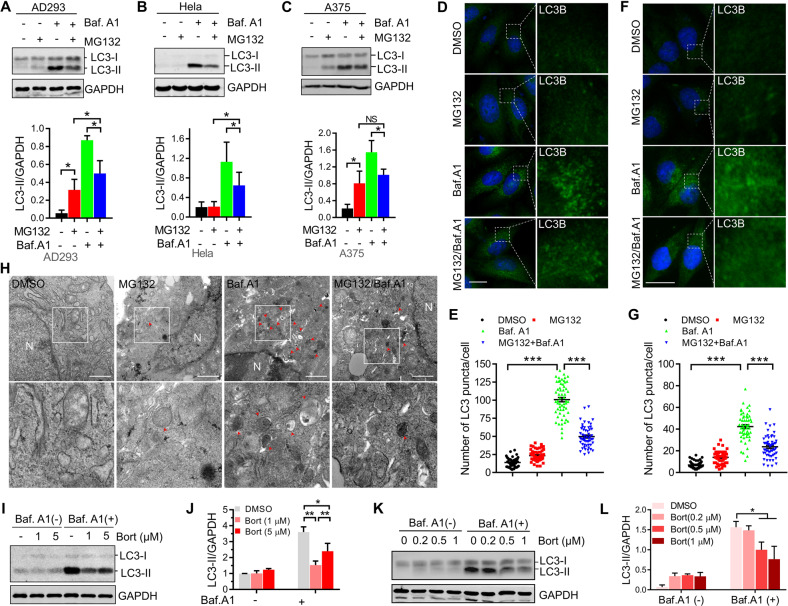
Suppressing autophagosome formation is the early response of cells to proteasome inhibition. **A**–**C** AD293, HeLa and A375 cells were treated with DMSO (control), or MG132 (2 μM), and Bafilomycin A1 (25 nM), alone or in combination for 14 h. The whole-cell lysates were subjected to western blot analysis with indicated antibodies. **D** HeLa cells were treated with DMSO (control), or MG132 (1 μM), and Bafilomycin A1 (25 nM), alone or in combination for 14 h. The LC3B-positive autophagosomes were analyzed by confocal microscopy. Scale bar: 10 μm. **E** Quantitative analysis of the results in (**D**). **F** A375 cells were treated with DMSO (control), or MG132 (2 μM), and Bafilomycin A1 (25 nM), alone or in combination for 14 h. The LC3B-positive autophagosomes were analyzed by confocal microscopy. Scale bar: 10 μm. **G** Quantitative analysis of the results in (**F**). **H** Representative electron micrographs from A375 cells treated with DMSO (control), or MG132 (2 μM), and Bafilomycin A1 (25 nM), alone or in combination for 14 h. N, nucleus; Arrows, autophagosomes or autolysosomes; Scale bar: 1 μm. **I** HeLa cells were treated with Bortezomib at indicated concentrations with or without 25 nM Bafilomycin A1 for 14 h, and then the whole-cell lysates were subjected to western blot analysis with indicated antibodies. **J** Quantitative analysis of the results in (**I**). **K** A375 cells were treated with Bortezomib at indicated concentrations with or without 25 nM Bafilomycin A1 for 14 h, and then the whole-cell lysates were subjected to western blot analysis with indicated antibodies. **L** Quantitative analysis of the results in (**K**). For **A**, **B**, **C**, **J**, and **L**, data are mean ± SEM of three independent experiments. *=*P* < 0.05, **=*P* < 0.01, NS = not significant. For **E** and **G**, at least 50 cells from three independent experiments were scored for each group. Data are mean ± SEM; ***P < 0.001.

To elucidate how proteasome inhibition alters autophagy, we tested the levels of LC3-II, an autophagosome marker [35], in cells treated with low concentration (2 μM) of the proteasome inhibitor, MG132, for 14 h. Interestingly, our western blot results showed that when the degradation of autophagosome was inhibited by Bafilomycin A1, a chemical that inhibits the maturation of autolysosome and the degradation of autophagic substances, proteasome inhibition dramatically reduced LC3B-II protein levels in AD293, HeLa and A375 cells (Fig. 1A–C), suggesting that autophagosome formation was promptly blocked after proteasome inhibition. Similar results were also found when lysosome activity was inhibited with Chloroquine (Supplementary Fig. S1A). To confirm the above finding, we also examined the autophagosome level in HeLa and A375 cells through immunostaining LC3B and observed that proteasome impairment significantly suppressed autophagosome formation (Fig. 1D–G). Similar results were found in A375 cells by transmission electronic microscopy (Fig. 1H). To exclude any off-target effects of MG132 on autophagy, we used another chemical, Bortezomib, to suppress the proteasome. Similar to MG132, a low concentration of Bortezomib was added to cells for a 14-h incubation, and we observed a reduction of LC3-II levels in A375 and HeLa cells following autophagic flux inhibition using Bafilomycin A1 (Fig. 1I–L). Since inhibiting proteasomes reduced the number of the autophagosome, we next investigated the initiation of autophagy by measuring the autophagy initiation proteins, such as Beclin1, WIPI2, ATG5, and ATG16L1. Interestingly, we observed that proteasome inhibition didn’t affect the expression of these proteins and the formation of the phagophore, a double membrane structure and maturing into autophagosome, by immunostaining WIPI2 and ATG5 (Supplementary Fig. S1B–D). These results suggest that the regulation of autophagy by proteasome inhibition mainly affects the later stage of autophagosome maturation, rather than the initiation. Sha et al. previously demonstrated that prolonged proteasome inhibition causes the induction of autophagy gene expression in myeloma and neuroblastoma cells [12]. To that end, we tested the effect of different proteasome inhibitor concentrations or treatment times on the LC3B-II levels. The data showed that increasing the dose or prolonging the treatment time of proteasome inhibitor enhanced LC3-II protein levels (Supplementary Fig. S1E, F), suggesting that the severe suppression of proteasome activity could elevate autophagosome formation. Taken together, these results indicate that the early stage of proteasome inhibition could suppress autophagosome formation.

### Proteasome inhibition cannot induce the autophagic degradation of polyubiquitinated proteins

**Fig. 2:**
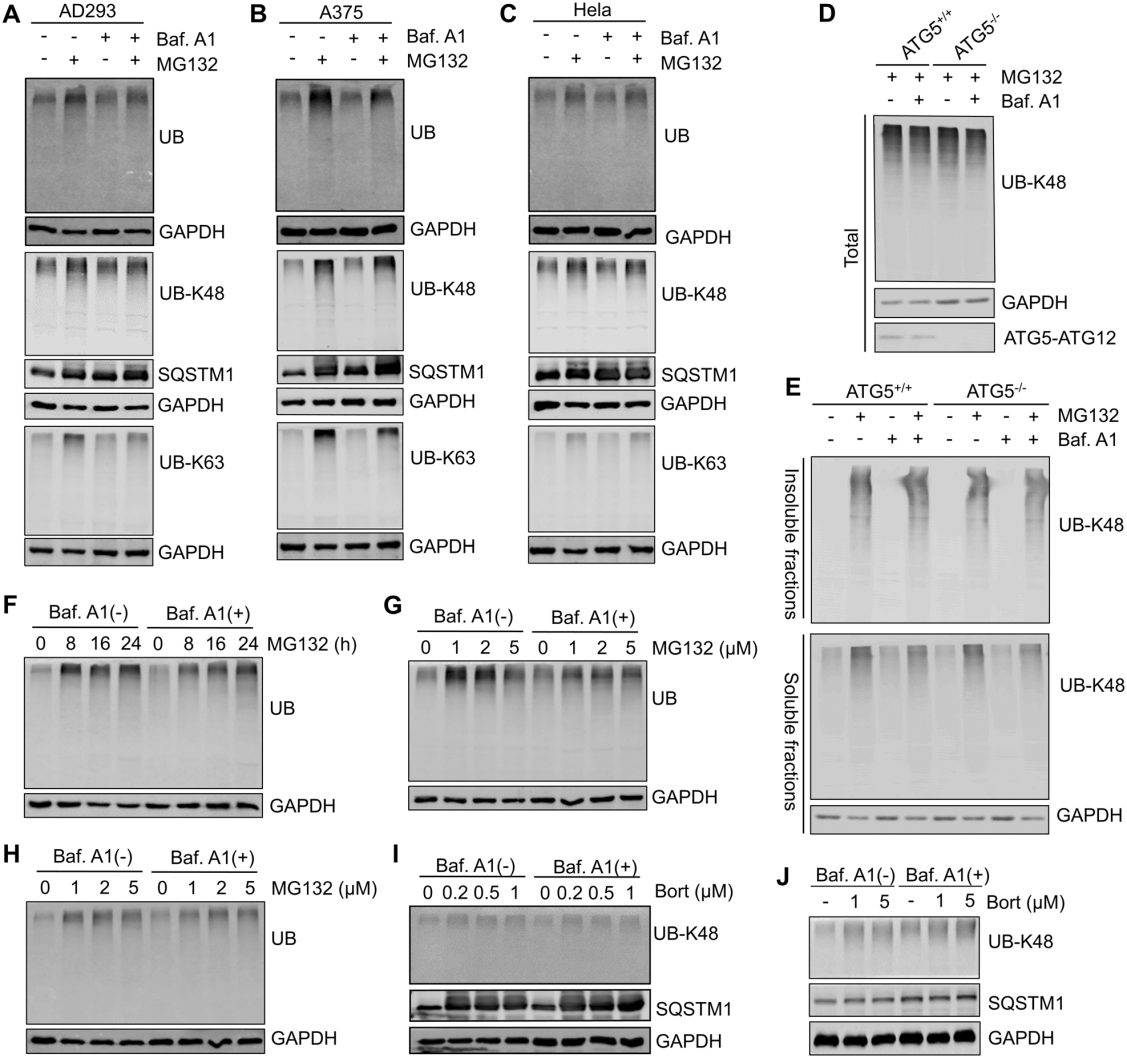
Proteasome inhibition cannot induce the autophagic degradation of polyubiquitinated proteins. **A**–**C** AD293, HeLa, and A375 cells were treated with DMSO (control), or MG132 (2 μM), and Bafilomycin A1 (25 nM), alone or in combination for 14 h. The whole-cell lysates were subjected to western blot analysis with indicated antibodies. **D**, **E** Wild-type (ATG5^+/+^) or ATG5 knockout (ATG5^−/−^) AD293 cells were treated with DMSO (control), or MG132 (2 μM), and Bafilomycin A1 (25 nM), alone or in combination for 14 h. The whole-cell lysates (Total), NP-40-soluble and -insoluble fractions were subjected to western blot analysis with indicated antibodies. **F** AD293 cells were treated with MG132 (2 μM) for the indicated time with or without Bafilomycin A1 (25 nM). The whole-cell lysates were subjected to western blot analysis with indicated antibodies. **G**, **H** AD293 cells (**G**) and A375 cells (**H**) were treated with MG132 at indicated concentrations with or without Bafilomycin A1 for 14 h (25 nM), and then the whole-cell lysates were subjected to western blot analysis with indicated antibodies. **I**, **J** A375 cells (**I**) and HeLa cells (**J**) were treated with Bortezomib at indicated concentrations with or without 25 nM Bafilomycin A1 for 14 h, and then the whole-cell lysates were subjected to western blot analysis with indicated antibodies.

Next, we investigated the autophagic degradation of polyubiquitinated proteins during proteasome inhibition. Although SQSTM1 showed a more marked increase in cells co-treated with MG132 and Bafilomycin A1, polyubiquitinated proteins, including K48-linked polyubiquitinated proteins (UB-K48) and K63-linked polyubiquitinated proteins (UB-K63), which are two main types of ubiquitinated proteins recruited into autophagic degradation, did not accumulate compared to cells treated with MG132 alone (Fig. 2A–C). This is in line with previous studies reporting no increase in lysosomal protein degradation upon proteasome inhibition [12, 36]. In addition, we found that the knockout of Autophagy protein 5 (ATG5) in AD293 cells did not induce an increase of polyubiquitinated proteins in both whole-cell lysates and the NP-40-insoluble fractions (Fig. 2D, E), in which protein aggregates accumulate due to they can’t be solubilized by mild detergent [8, 37]. Interestingly, although the autophagy flux was upregulated upon extensive proteasome activity inhibition, we did not observe autophagic degradation of polyubiquitinated proteins due to the failed accumulation of polyubiquitinated proteins in response to Bafilomycin A1 treatment (Fig. 2F–H). Similar results were observed in Bortezomib-treated cells (Fig. 2I, J). Taken together, these results indicate that autophagic degradation of polyubiquitinated proteins was not among the consequences of cells responding to proteasome inhibition.

### Suppressing autophagy promotes the aggresome formation during proteasome inhibition

**Fig. 3:**
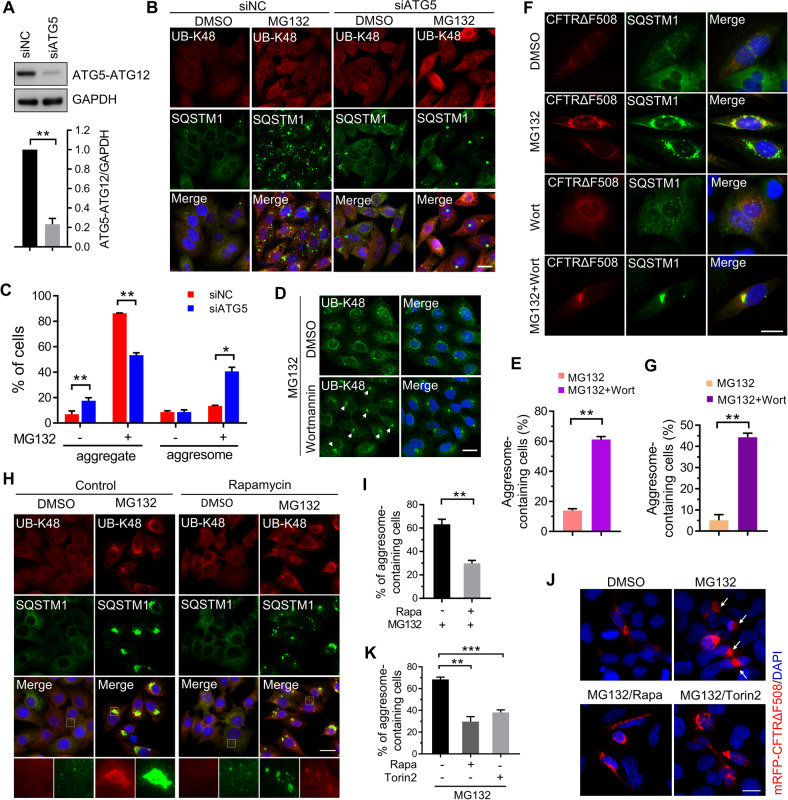
Blocking autophagy promotes proteasome inhibitor-induced aggresome formation. **A** HeLa cells were transfected with siRNA targeting ATG5 (siATG5) or control siRNA (siNC) for 48 h. The efficiency of ATG5 knockdown was analyzed with western blot using anti-ATG5 and anti-GAPDH antibodies. Quantitative data are mean ± SEM of three independent experiments. **P < 0.01. **B** HeLa cells were transfected with siRNA targeting ATG5 (siATG5) or control siRNA (siNC) for 48 h, and then treated with DMSO or 1 μM MG132 for 14 h. The aggresome formation was analyzed by immunostaining with anti-UB-K48 (Red) and anti-SQSTM1 (Green) antibodies. Nuclei were stained with DAPI (blue). Scale bar: 20 μm. **C** Quantitative analysis of results in (**C**). **D** HeLa cells were treated with MG132 (1 μM), or MG132 (1 μM)/Wortmannin (5 μM) for 14 h. The aggresome formation was analyzed by immunostaining with anti-UB-K48 (Green). Nuclei are stained with DAPI (blue). Scale bar: 20 μm. **E** Quantitative analysis of results in (**E**). **F** HeLa cells were transfected with plasmids expressing mRFP-CFTR∆F508 for 24 h, and then treated with DMSO (control), or MG132 (1 μM), or Wortmannin (5 μM), or MG132 (1 μM)/ Wortmannin (5 μM) for 14 h. The aggresome formation was analyzed by immunostaining with anti-SQSTM1 (Green) antibodies. Nuclei were stained with DAPI (blue). Scale bar: 10 μm. **G** Quantitative analysis of results in (**F**). **H** A375 cells with or without 2 μM Rapamycin pretreatment for 10 h were treated with DMSO (control) or MG132 (2 μM) for 14 h. The aggresome formation was analyzed by immunostaining with anti-UB-K48 (red) and anti-SQSTM1 (green) antibodies. Nuclei were stained with DAPI (blue). Scale bar: 20 μm. **I** Quantitative analysis of results in (**H**). **J** AD293 cells were transfected with plasmids expressing mRFP-CFTR∆F508 for 24 h, and then treated with DMSO (control), or MG132 (2 μM), or MG132 (2 μM)/ Torin 2 (1 μM) for 14 h, or pretreated with Rapamycin (2 μM) for 10 h and then treated with MG132 (2 μM)/Rapamycin (2 μM) for 14 h. The aggresome formation of mRFP-CFTR∆F508 was analyzed by fluorescence microscope. Nuclei were stained with DAPI (blue). Scale bar: 20 μm. **K** Quantitative analysis of results in (**J**). For **C**, **E**, **G**, **I**, and **K**, at least 50 cells were randomly selected from each group to score for aggresomes. Data are mean ± SEM of three independent experiments. *P < 0.05, **P < 0.01, ***P < 0.001.

Our previous studies have demonstrated that phosphorylation of SQSTM1 T269/S272 promoted the aggresome formation upon proteasome inhibition [34]. In addition, we also found in HeLa cells, ubiquitinated proteins rarely formed aggresomes upon proteasome inhibition due to the insufficient phosphorylation of SQSTM1 T269/S272 (Supplementary Fig. S2). Next, we tested whether autophagy affects the aggresome formation in HeLa cells. We blocked the autophagy of HeLa cells by transfecting siRNA targeting ATG5 (Fig. 3A). After being treated with 1 μM MG132 for 14 h, only 13.48% of the control cells formed perinuclear aggresome marked with UB-K48 and SQSTM1, while the percentage in ATG5 knockdown cells rose to 40.66% (Fig. 3B, C). Moreover, we observed that during proteasome suppression, inhibiting autophagy with Wortmannin, an inhibitor of autophagosome initiation, dramatically elevated the aggresome formation of polyubiquitinated proteins in HeLa cells (Fig. 3D, E). Although UB-K48 and SQSTM1 could well indicate aggresome structure [29, 38], we further confirmed the aggresome structure by other aggresome markers, HDAC6 and γ-tubulin [7, 8]. As expected, the perinuclear inclusion body formed by UB-K48 and SQSTM1 strongly overlapped with HDAC6 and γ-tubulin (Supplementary Fig. S3A–C). In addition, we expressed mRFP tagged CFTRΔF508, a CFTR mutant normally degraded by the proteasome and recruited to the aggresome during proteasome dysfunction [7], in HeLa cells. Similar results were observed whereby autophagy inhibition promoted misfolded mRFP-CFTRΔF508 to form aggresomes (Fig. 3F, G). Interestingly, knockout of ATG5 in AD293 cells, which reportedly strongly form aggresomes when proteasome activity is inhibited [29, 34], did not influence the rate and size of polyubiquitinated proteins aggresome formation (Supplementary Fig. S3D–F), suggesting the presence of another mechanism that could eliminate the effect of autophagy on aggresome formation. Since inhibition of autophagy has no effects on aggresome formation in cells that strongly form aggresome in response to proteasome inhibition, we examined whether elevating the autophagy flux could suppress aggresome biogenesis in those cells. Compared with cells treated with MG132 alone, cells pretreated with Rapamycin, an mTOR inhibitor that actives autophagosome initiation and maturation [39], showed a significantly reduced aggresome formation in A375 cells (Fig. 3H, I and Supplementary Fig. S3G), which is consistent with the findings found in MEF cells by Zhou et al. [40]. Interestingly, instead of being concentrated into aggresome, SQSTM1 and UB-K48 were distributed as micro-aggregates in autophagy-activated cells (Fig. 3H). Moreover, we also observed that rapamycin or Torin 2, another mTOR inhibitor, strongly inhibited the aggresome formation of mRFP-CFTRΔF508 expressed in AD293 cells (Fig. 3J, K). We also found that high-dose treatment by proteasome inhibitor impaired the aggresome formation of polyubiquitinated proteins in A375 cells (Supplementary Fig. S3H, I). Collectively, these data suggest that suppressing autophagy promotes aggresome formation during proteasome inhibition.

### Doramapimod prevents aggresome formation via inhibiting the phosphorylation of SQSTM1 (Thr269/Ser272)

**Fig. 4:**
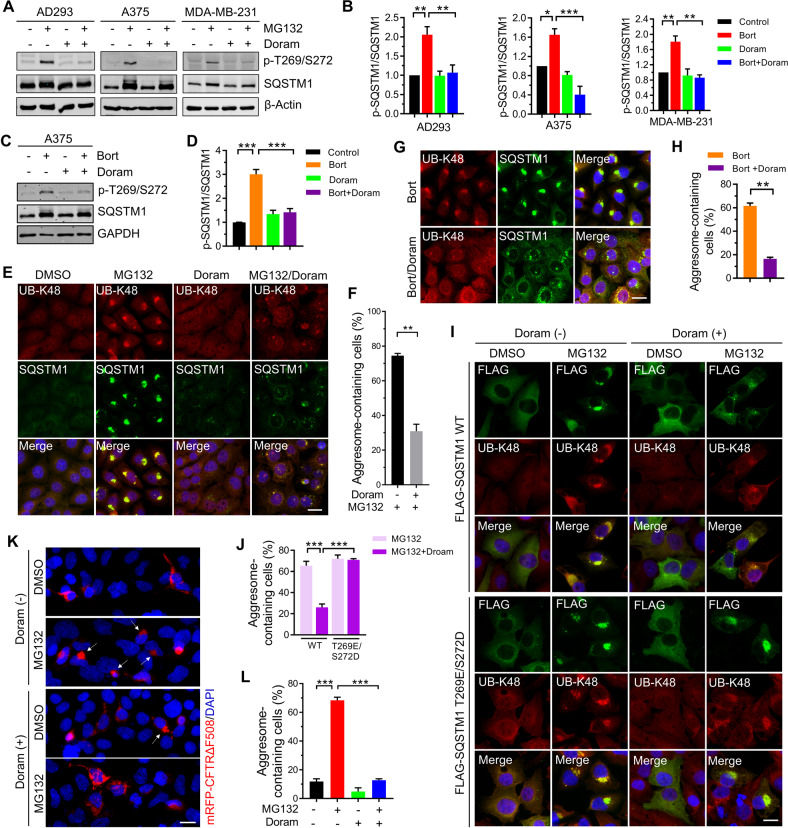
Doramapimod prevents the aggresome formation via inhibiting the phosphorylation of SQSTM1(Thr269/Ser272). **A** AD293, A375 and MDA-MB-231 cells were treated with DMSO (control), or MG132 (2 μM), and Doramapimod (50 μM), alone or in combination for 14 h. The whole-cell lysates were subjected to western blot analysis with indicated antibodies. **B** Quantitative analysis of results in (**A**). Data are mean ± SEM of three independent experiments. *P < 0.05, **P < 0.01, ***P < 0.001. **C** A375 cells were treated with DMSO (control), or Bortezomib (1 μM), and Doramapimod (50 μM), alone or in combination for 14 h. The whole-cell lysates were subjected to western blot analysis with indicated antibodies. **D** Quantitative analysis of results in (**C**). Data are mean ± SEM of 3 independent experiments. *P < 0.05, **P < 0.01. **E** A375 cells were treated with DMSO (control), or MG132 (2 μM), and Doramapimod (50 μM), alone or in combination for 14 h. The aggresome formation was analyzed by immunostaining with anti-UB-K48 (Red) and anti-SQSTM1 (Green) antibodies. Nuclei were stained with DAPI (blue). Scale bar: 20 μm. **F** Quantitative analysis of results in (**E**). **G** A375 cells were treated with Bortezomib (1 μM), or Bortezomib (1 μM)/Doramapimod (50 μM) for 14 h. The aggresome formation was analyzed by immunostaining with anti-UB-K48 (red) and anti-SQSTM1 (green) antibodies. Nuclei were stained with DAPI (blue). Scale bar: 20 μm. **H** Quantitative analysis of results in (**G**). **I** A375 cells were transfected with plasmids expressing FLAG-SQSTM1(WT) or FLAG-SQSTM1(T269E/S272D) for 24 h, and then treated with DMSO (control), or MG132 (2 μM), and Doramapimod (50 μM), alone all in combination for 14 h. The aggresome formation was analyzed by immunostaining with anti-UB-K48 (Red) and anti-FLAG (green) antibodies. Nuclei were stained with DAPI (blue). Scale bar: 20 μm. **J** Quantitative analysis of results in (**I**). **K** AD293 cells were transfected with plasmids expressing mRFP-CFTR∆F508 for 24 h, and then treated with DMSO (control), or MG132 (2 μM), and Doramapimod (50 μM), alone all in combination for 14 h. The aggresome formation was analyzed by fluorescence microscope. Nuclei were stained with DAPI (blue). Scale bar: 20 μm. **L** Quantitative analysis of results in (**K**). For **F**, **H**, **J**, and **L**, at least 40 cells were randomly selected from each group to score for aggresomes. Data are mean ± SEM of three independent experiments. **P < 0.01, ***P < 0.001.

Our previous studies have demonstrated that the phosphorylation of SQSTM1 at Thr269/Ser272 (T269/S272) is critical for the aggresome formation of misfolded protein in AD293 cells [34]. Since it was previously reported that p38γ/p38δ, which are activated upon proteasome inhibition [34], could phosphorylate SQSTM1 at T269/S272 [34, 41, 42], we tested whether suppressing p38γ/p38δ activity could decrease SQSTM1 (T269/S272) phosphorylation and aggresome biogenesis. Western blot assay showed that Doramapimod, a chemical that inhibits p38 MAPKs specifically, significantly reduced the level of SQSTM1 (T269/S272) phosphorylation induced by MG132 in AD293, A375, and MDA-MB-231 cells (Fig. 4A, B and Supplementary Fig. S4A). Similarly, Bortezomib-induced T269/S272 phosphorylation was also inhibited by Doramapimod (Fig. 4C, D). Next, we examined the aggresome formation in cells treated with Doramapimod alone or in combination with proteasome inhibitors. As shown in Fig. 4E–H, Doramapimod significantly reduced the aggresome formation rates upon proteasome inhibition in A375 cells. Apart from p38γ/p38δ, Doramapimod could also inhibit the kinase activity of two other p38 MAPKs, p38α and p38β. To exclude the possibility that Doramapimod might inhibit aggresome formation by suppressing the kinase activity of p38α/p38β, we used p38α/p38β-specific inhibitor, SB203580, and SB202190, to treat cells. We revealed that SB203580 and SB202190 did not reduce the phosphorylation of SQSTM1(T269/S272) and the aggresome formation following proteasome inhibition (Supplementary Fig. S4B, C). Besides, it has been reported that Doramapimod could inhibit Abl and BRAF [43]. Thus, we tested the effect of Abl and BRAF on proteasome inhibition-induced aggresome formation with their specific inhibitors. We found that inhibition of Abl or BRAF did not affect the phosphorylation of SQSTM1(T269/S272) and the aggresome formation upon proteasome inhibition (Supplementary Fig. S4D, E). These results suggest that p38γ/p38δ could be the targets of Doramapimod in aggresome biogenesis regulation. To confirm that the defect of aggresome formation caused by Doramapimod was dependent on failed SQSTM1 (T269/S272) phosphorylation, we examined whether the phosphomimetic (T269E/S272D) mutant of SQSTM1 could rescue the aggresome formation defect. The immunostaining results showed that expression of SQSTM1 (T269E/S272D) mutants but not SQSTM1 (WT) successfully rescued Doramapimod-mediated aggresome formation defect (Fig. 4I, J), suggesting that Doramapimod could prevent aggresome formation via inhibiting the phosphorylation of SQSTM1 (T269/S272). In addition, we expressed mRFP-CFTRΔF508 in AD293 cells and observed Doramapimod also reduced the aggresome formation of mRFP-CFTRΔF508 (Fig. 4K, L). Taken together, these results indicate that Doramapimod could prevent aggresome formation via inhibiting the phosphorylation of SQSTM1 (Thr269/Ser272).

### Suppressing autophagy rescues the defective aggresome formation caused by unphosphorylated SQSTM1 (T269/S272)

**Fig. 5:**
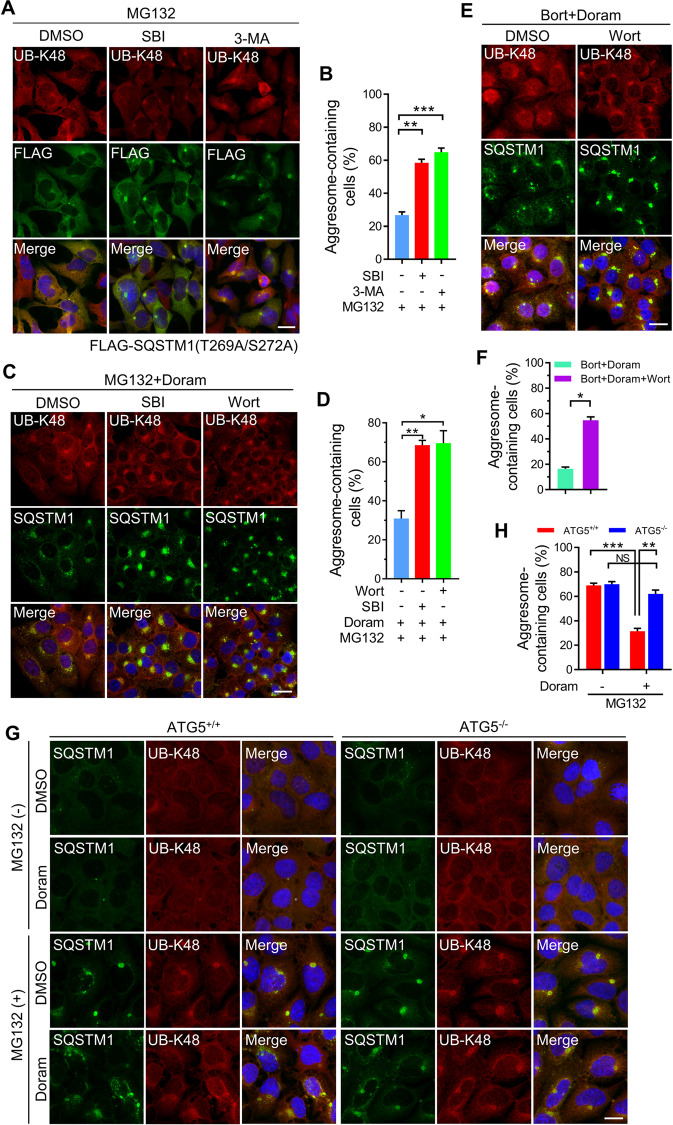
Blocking autophagy rescues the defective aggresome formation caused by the failure of SQSTM1(T269/S272) phosphorylation. **A** SQSTM1 knockout AD293 cells stably re-expressing FLAG-SQSTM1(T269A/S272A) were treated with MG132 (2 μM), or MG132 (2 μM)/SBI-0206965 (5 μM), or MG132 (2 μM)/3-MA (100 μM) for 14 h. The aggresome formation was analyzed by immunostaining with anti-UB-K48 (Red) and anti-FLAG (Green) antibodies. Nuclei were stained with DAPI (blue). Scale bar: 20 μm. **B** Quantitative analysis of results in (**A**). **C** A375 cells were treated with MG132 (2 μM)/Doramapimod (50 μM), or MG132 (2 μM)/ Doramapimod (50 μM)/SBI-0206965 (5 μM), or MG132 (2 μM)/Doramapimod (50 μM)/Wortmannin (5 μM) for 14 h. The aggresome formation was analyzed by immunostaining with anti-UB-K48 (red) and anti-SQSTM1 (green) antibodies. Nuclei were stained with DAPI (blue). Scale bar: 20 μm. **D** Quantitative analysis of results in (**C**). **E** A375 cells were treated with Bortezomib (1 μM)/Doramapimod (50 μM), or Bortezomib (1 μM)/Doramapimod (50 μM)/Wortmannin (5 μM) for 14 h. The aggresome formation was analyzed by immunostaining with anti-UB-K48 (red) and anti-SQSTM1 (green) antibodies. Nuclei were stained with DAPI (blue). Scale bar: 20 μm. **F** Quantitative analysis of results in (**E**). **G** Wild-type (ATG5^+/+^) or ATG5 knockout (ATG5^−/−^) AD293 cells were treated with DMSO (control), or MG132 (2 μM), or Doramapimod (50 μM), or MG132 (2 μM)/Doramapimod (50 μM) for 14 h. The aggresome formation was analyzed by immunostaining with anti-UB-K48 (red) and anti-SQSTM1 (green) antibodies. Nuclei were stained with DAPI (blue). Scale bar: 20 μm. **H** Quantitative analysis of results in (**G**). For **B**, **D**, **F**, and **H**, at least 50 cells were randomly selected from each group to score for aggresomes. Data are mean ± SEM of three independent experiments. *P < 0.05, **P < 0.01, ***P < 0.001, NS = not significant.

We observed that neither SQSTM1 (T269A/S272A) overexpression nor Doramapimod treatment affected LC3-II protein level when proteasome activity was inhibited (Supplementary Fig. S5A, B), suggesting that SQSTM1(T269/S272) phosphorylation did not take part in the regulation of autophagy. Then we tested whether inhibiting autophagy may rescue aggresome formation defect in cells expressing SQSTM1 (T269A/S272A). Surprisingly, we found that inhibiting autophagy using small molecule kinase inhibitor SBI-0206965, a specific inhibitor of ULK1/2, or 3-Methyladenine (3-MA), a PI3K inhibitor, significantly increased the aggresome formation rates in SQSTM1 knockout (SQSTM^−/−^) cells stably re-expressing SQSTM1(T269A/S272A) (Fig. 5A, B), which has been reported to suppress aggresome biogenesis. Similar results were also observed in HeLa cells stably expressing SQSTM1 (T269A/S272A) (Supplementary Fig. S5C, D). Besides, we treated A375 cells using a combination of proteasome inhibitors, Doramapimod, and autophagy inhibitors. The data showed that inhibiting autophagy using SBI-0206965 or Wortmannin elevated the rates of aggresome formation of A375 cells following co-treated with MG132 or Bortezomib and Doramapimod (Fig. 5C–F). Similarly, Wortmannin also rescued the Doramapimod-mediated aggresome formation defect of MDA-MB-231 cells (Supplementary Fig. S5E and F). In addition, we examined the aggresome formation of ATG5 knockout AD293 cells and found that the knockout of ATG5 significantly rescued the defective aggresome formation caused by Doramapimod (Fig. 5G, H). Collectively, these data indicate that blocking autophagy could rescue the defect of aggresome formation caused by failed SQSTM1 (T269/S272) phosphorylation during proteasome impairment.

### SQSTM1(T269/S272) phosphorylation inhibits its autophagic receptor activity upon proteasome inhibition

**Fig. 6:**
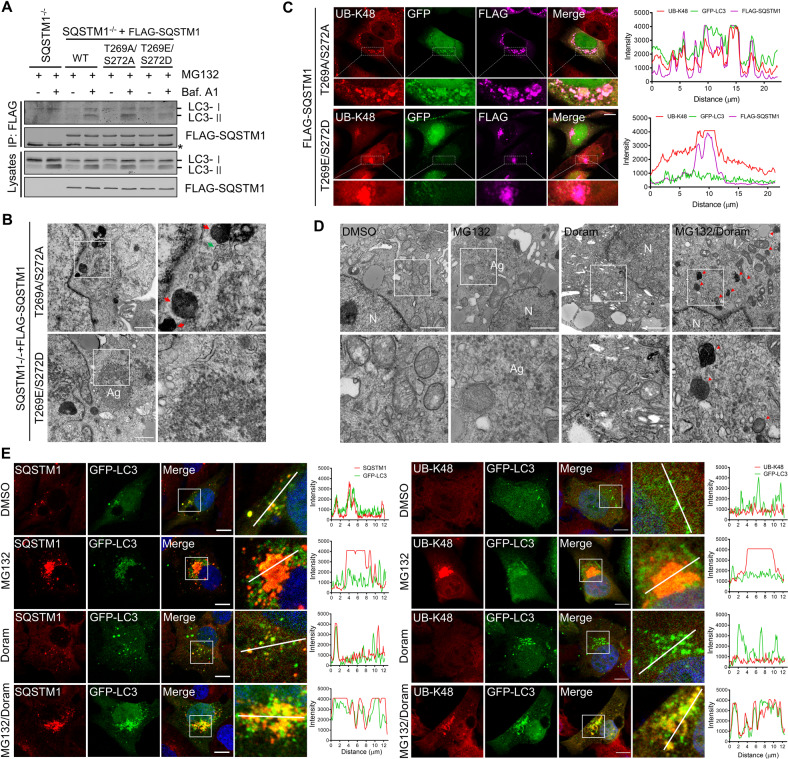
SQSTM1(T269/S272) phosphorylation inhibits its autophagic receptor activity upon proteasome inhibition. **A** SQSTM1^−/−^ and its rescue cell lines were treated with MG132 (10 μM) with or without Bafilomycin A1 (25 nM) for 5 h. Cell lysates were immunoprecipitated with anti-FLAG antibodies. Co-immunoprecipitated proteins were detected by western blot analysis using indicated antibodies. Asterisks indicate IgG. **B** Representative electron micrographs from SQSTM1^−/−^ cell lines stably expressing SQSTM1 mutants after treated with MG132 (2 μM) for 14 h. N, nucleus; Arrows, sequestered aggregates; Ag, aggresome; Scale bar: 1 μm. **C** HeLa cells stably expressing EGFP-LC3B were transfected with plasmids expressing FLAG-SQSTM1(T269A/S272A) or FLAG-SQSTM1(T269E/S272D) for 24 h, and then treated with MG132 (1 μM) and Bafilomycin A1 (25 nM) for 14 h. The cells were then fixed and analyzed by immunostaining with anti-UB-k48 (red) and anti-FLAG (green) antibodies. Nuclei were stained with DAPI (blue). Scale bar: 10 μm. The fluorescence intensity line is tracing corresponding to a white line. **D** Representative electron micrographs from A375 cells after treated with indicated inhibitors (MG132 (2 μM), Doramapimod (50 μM)) for 14 h. N, nucleus; Arrows, sequestered aggregates; Ag, aggresome; Scale bar: 1 μm. **E** A375 cells were treated with indicated inhibitors (MG132 (2 μM), Doramapimod (50 μM)) for 14 h. Cells were then fixed and immunostained with anti-SQSTM1 or anti-UB-K48 antibodies. Nuclei were stained with DAPI (blue). Scale bar: 10 μm. The fluorescence intensity line is tracing corresponding to a white line.

Previous studies have reported that SQSTM1 phosphorylation could block autophagosome-associated recruitment [44]. Given our finding that autophagy inhibition could rescue the aggresome formation caused by failed SQSTM1(T269/S272) phosphorylation, we expected SQSTM1(T269/S272) phosphorylation would prevent its recruitment to the autophagosome. It has been reported that self-oligomerization and LC3 binding are main recruitment mechanisms for SQSTM1 targeting to autophagosome [28, 45]. We next analyzed the self-oligomerization of SQSTM1 by co-immunoprecipitation experiment and found the failure of SQSTM1(T269/S272) phosphorylation did not influence the self-oligomerization (Supplementary Fig. S6A). Next, we examined the interaction between SQSTM1 and LC3B. We found that compared with SQSTM1 (WT) or SQSTM1 (T269E/S272D), SQSTM1 (T269A/S272A) formed a stronger bond with LC3B proteins in cells that were treated with MG132 and bafilomycin A1 (Fig. 6A). Moreover, we co-expressed FLAG-LC3B, Myc-SQSTM1 and FLAG-p38δ (wild-type (WT), kinase-dead mutant (K54R), or constitutively active mutant (F324S)). The data showed that co-expressed p38δ(WT) and p38δ(F324S) dramatically reduced SQSTM1-associated LC3B proteins (Supplementary Fig. S6B). Although the expression of p38δ(K54R) also induced a reduction in SQSTM1 and LC3B interactions, it elevated the amount of SQSTM1-bound LC3B compared with p38δ(WT) or p38δ(F324S) (Supplementary Fig. S6B). Taken together, these results suggest that SQSTM1 (T269/S272) phosphorylation could inhibit its interaction with LC3B. Next, we examined whether SQSTM1 (T269/S272) phosphorylation inhibits the autophagic recruitment of SQSTM1 and its associated polyubiquitinated proteins. Electron microscopy revealed that in SQSTM1^−/−^ cells stably expressing T269E/S272D, distinct large membrane-free, aggresome-like structures could be formed in the juxtanuclear region. While in SQSTM1^−/−^ cells stably expressing T269A/S272A, these aggregate-like structures were dispersed and enclosed by membranes (Fig. 6B). In addition, we found that upon proteasome and autophagy flux inhibition in HeLa cells, most of the SQSTM1 (T269E/S272D) was concentrated in the inclusion bodies near nuclear with UB-K48, where GFP-LC3B is absent (Fig. 6C). In contrast, SQSTM1(T269A/S272A) was distributed as micro-aggregates and co-localized with GFP-LC3B and UB-K48 (Fig. 6C). Moreover, with electron microscopy analysis, we found that Doramapimod inhibited the aggresome-like structures formation but induced the increase of aggregate-like structures enclosed by the membrane in A375 cells (Fig. 6D). We also found that in alone proteasome inhibitor-treated A375 cells SQSTM1 and UB-K48 mainly formed the unique aggresome without LC3B puncta that clustering in the prenuclear region around the aggresome (Fig. 6E). However, in cells exposed to Doramapimod and proteasome inhibitors, SQSTM1 and UB-K48 presented as puncta and mainly co-stained with LC3B (Fig. 6E). Collectively, these data indicate that SQSTM1 (T269/S272) phosphorylation could prevent its delivery to the autophagosome in response to proteasomal inhibition.

### Non-phosphorylation of SQSTM1 (T269/S272) does not enhance the autophagic degradation of polyubiquitinated proteins during proteasome inhibition

**Fig. 7:**
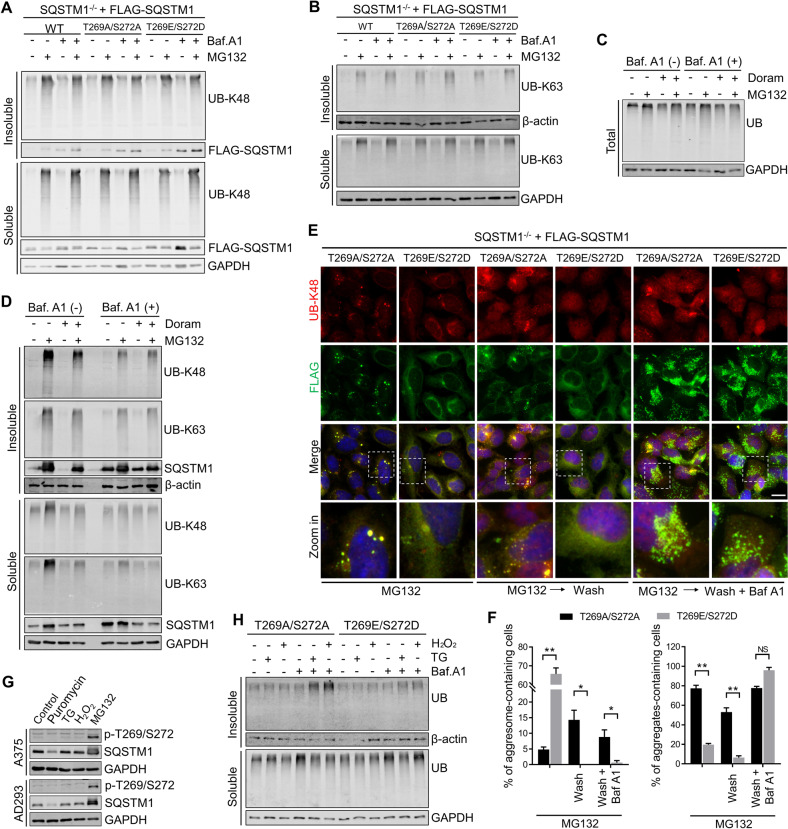
Non-phosphorylation of SQSTM1 (T269/S272) does not enhance the autophagic degradation of polyubiquitinated proteins during proteasome inhibition. **A**, **B** SQSTM1^−/−^ cells stably expressing indicated constructs were treated with DMSO (control), or MG132 (2 μM), and Bafilomycin A1 (50 nM), alone or in combination for 12 h. Whole-cell extracts were separated into NP-40-soluble and -insoluble fractions and subjected to western blot analysis with indicated antibodies. **C**, **D** A375 cells were treated with indicated inhibitors (MG132 (2 μM), Bafilomycin A1 (25 nM), and Doramapimod (50 μM)) for 14 h. The whole-cell lysates (Total), NP-40-soluble, and -insoluble fractions were subjected to western blot analysis with indicated antibodies. **E** SQSTM1^−/−^ cells stably expressing indicated constructs were treated with MG132 (2 μM) for 12 h, then media were switched to fresh culture media with or without Bafilomycin A1 (20 nM) for 24 h. Cells were fixed and immunostained with anti-UB-K48 (Red) and anti-FLAG (green) antibodies. Nuclei were stained with DAPI (blue). Scale bar: 20 μm. **F** Quantitative analysis of results in (**E**). Data are mean ± SD of three independent experiments. For each experiment, at least 50 cells were randomly selected from each group to score *P < 0.05, **P < 0.01, NS = not significant. **G** A375 and AD293 cells were treated with Puromycin (2 μg/mL), Thapsigargin (5 μg/mL), H_2_O_2_ (200 μM), or MG132 (1 μm) for 24 h. the whole-cell lysates were then subjected to western blot analysis with indicated antibodies. **H** SQSTM1^−/−^ cells stably expressing indicated constructs were treated with DMSO (control), or Thapsigargin (5 μg/mL), H_2_O_2_ (200 μM), and Bafilomycin A1 (50 nM), alone or in combination for 24 h. Whole-cell extracts were separated into NP-40-soluble and -insoluble fractions and subjected to western blot analysis with indicated antibodies.

Since non-phosphorylated SQSTM1 (T269/S272) displayed a stronger association with the autophagosome, we subsequently examined whether it could promote the autophagic degradation of ubiquitinated proteins. Interestingly, we did not observe increased accumulation of NP-40 -insoluble polyubiquitinated proteins in SQSTM1^−/−^ cells re-expressing SQSTM1 (T269A/S272A) following co-treatment with MG132 and Bafilomycin A1, compared with the cells re-expressing SQSTM1 (WT) or SQSTM1 (T269E/S272D) (Fig. 7A, B). Consistently, Doramapimod did not enhance the autophagic degradation of polyubiquitinated proteins in A375 cells (Fig. 7C, D). Taken together, these results further indicate that autophagic degradation of SQSTM1-associated polyubiquitinated proteins is suppressed during proteasome inhibition.

It is well established that autophagic degradation is the main pathway for cells to eliminate the aggregates of polyubiquitinated proteins [46, 47]. Thus, we tested the effect of SQSTM1 mutants on the clearance of aggregated polyubiquitinated proteins. SQSTM1^−/−^ cells re-expressing SQSTM1 (T269A/S272A) or SQSTM1 (T269E/S272D) were treated with proteasome inhibitor for 12 h to allow for the accumulation and formation of aggregates/aggresomes. The cells were then exposed to fresh culture media with or without Bafilomycin A1. The immunostaining results showed that after 24 h MG132 washout, UB-K48-associated aggresomes were well cleared in SQSTM1^−/−^-SQSTM1(T269E/S272D) cells, which were successfully blocked by Bafilomycin A1 (Fig. 7E, F). In contrast, the aggregates formed in SQSTM1^−/−^-SQSTM1 (T269A/S272A) cells were not removed following MG132 washout (Fig. 7E, F). These data indicate that targeting of polyubiquitinated proteins to aggresome where reportedly lysosome around facilitates the autophagic degradation of the abnormal proteins, rather than being presented to autophagosome as micro-aggregates.

Moreover, we examined the effect of SQSTM1 T269/S272 phosphorylation on aggrephagy induced by other cellular stress. We didn’t find the increase of SQSTM1 T269/S272 phosphorylation in AD293 and A375 cells treated with puromycin, a protein synthesis inhibitor and inducing misfolded protein aggregation, thapsigargin, an ER stress inducer, or H_2_O_2_, an oxidative stress inducer (Fig. 7G). However, compared with the non-phosphorylatable mutant (T269A/S272A), the phosphomimetic (T269E/S272D) mutant significantly inhibited the autophagic degradation of aggregated ubiquitinated protein induced by thapsigargin and H_2_O_2_ (Fig. 7H). In addition, we also found that non-phosphorylation of SQSTM1 T269/S272 did not influence the aggresome formation of mutant huntingtin that could form aggresome independent of ubiquitination and proteasome inhibition [48] (Supplementary Fig. S7). This evidence suggests a different molecular mechanism of autophagic degradation of misfolded proteins regulated by SQSTM1 under normal and abnormal proteasome activity.

### Doramapimod aggravates proteasome inhibitor-induced cell death and tumor suppression

**Fig. 8:**
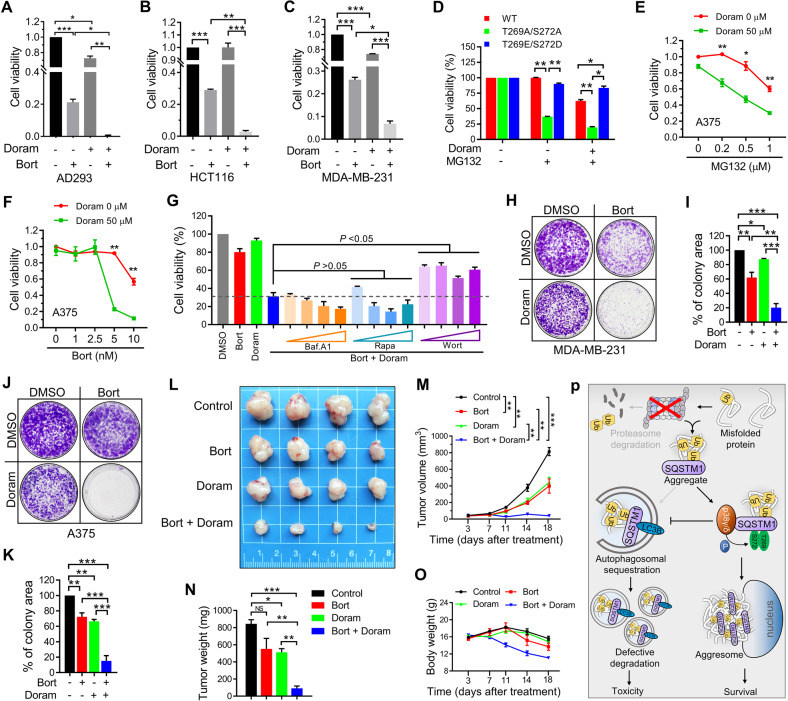
Doramapimod aggravates proteasome inhibitor-induced cell death and tumor suppression. **A** AD293 cells were treated with DMSO (control), or Bortezomib (20 nM), and Doramapimod (50 μM), alone or in combination for 36 h, and then examined the cell viability with CCK-8 assay. **B** HCT-116 cells were treated with DMSO (control), or Bortezomib (10 nM), and Doramapimod (50 μM), alone or in combination for 24 h, and then examined the cell viability with CCK-8 assay. **C** MDA-MB-231 cells were treated with DMSO (control), or Bortezomib (10 nM), and Doramapimod (50 μM), alone or in combination for 24 h, and then examined the cell viability with CCK-8 assay. **D** SQSTM1 knockout AD293 cells stably re-expressing FLAG-SQSTM1 (WT or mutants) were treated with DMSO (control), or MG132 (2 μM), or MG132 (2 μM)/Doramapimod (50 μM) for 36 h, and then examined the cell viability with CCK-8 assay. **E** A375 cells were treated with MG132 at indicated concentrations with or without Doramapimod (50 μM) for 24 h, and then examined the cell viability with CCK-8 assay. **F** A375 cells were treated with Bortezomib at indicated concentrations with or without Doramapimod (50 μM) for 48 h, and then examined the cell viability with CCK-8 assay. **G** A375 cells were treated with indicated inhibitors (Bortezomib (20 nM), Doramapimod (50 μM), Bafilomycin A1 (2, 5, 10, 20 nM), Rapamycin (0.2, 0.5, 1, 2 μM), Wortmannin (0.2, 0.5, 1, 2 μM)) for 24 h, and then examined the cell viability with CCK-8 assay. **H**, **J** MDA-MB-231 and A375 cells were treated with DMSO (control), or Bortezomib (5 nM), and Doramapimod (25 μM), alone or in combination for 7 days, and then analyzed the cell colony-forming ability. **I**, **K** Quantitative analysis of results in (**H**) and (**J**). **A**–**G**, **I**, **K** Data are mean ± SEM of three independent experiments. *P < 0.05, **P < 0.01, ***P < 0.001. **L**–**O** The anticancer effect of the combination of Bortezomib and Doramapimod in a A375 cell subcutaneous tumor model. **L** Excised tumors on day 18. **M** The tumor volume of each group was calculated two times every week. Data are mean ± SEM of four mice. **P < 0.01, ***P < 0.001. **N** The tumor weights of excised tumors on day 18. Data are mean ± SEM of four mice. *P < 0.05, **P < 0.01, ***P < 0.001, ****P < 0.0001, NS = not significant. **O** The body weight of each group was calculated two times every week. Data are mean ± SEM of four mice. **P** Model depicting proteasome inhibition-induced phosphorylation of SQSTM1 T269/S272 inhibits its autophagic receptor activity and promotes aggresome formation of misfolded proteins.

Several studies have confirmed that the sequestration of ubiquitinated proteins into aggresome protects against cell death caused by proteasome inhibition [7–9, 12, 34], which may mediate the drug resistance of tumor cells therapied with proteasome inhibitors [18, 49, 50]. Since Doramapimod abolishes the aggresome formation during proteasome inhibition, we wondered whether Doramapimod could enhance proteasome inhibitor-induced cell death. To test this, we analyzed the viability of cells treated with Bortezomib alone or with Doramapimod using a CCK-8 assay kit. In AD293, HCT-116, and MDA-MB-231 cells, we observed that the combination of the two drugs aggravated the loss of cell viability compared to a single treatment with Bortezomib (Fig. 8A–C). As demonstrated above, the defective aggresome formation mediated by Doramapimod is dependent on the failure of SQSTM1 (T269/S272) phosphorylation. Therefore, we surmised that SQSTM1 (T269E/S272D) should be capable of alleviating the cell damage caused by Doramapimod. As expected, in SQSTM^−/−^ AD293 cells, compared with SQSTM1 (WT) or SQSTM1 (T269A/S272A), re-expressing SQSTM1 (T269E/S272D) significantly elevated the cell viability when cells were treated with MG132 and Doramapimod (Fig. 8D). Moreover, Doramapimod reduced the resistance of A375 cells to both MG132 and Bortezomib (Fig. 8E, F). Subsequently, we tested whether inhibiting autophagy could rescue the cell death enhanced by SQSTM1 (T269A/S272A) or Doramapimod. We found blocking autophagy using Wortmannin, but not Bafilomycin A1, increased the cell viability of A375 cells during treated with Bortezomib and Doramapimod (Fig. 8G). Next, we analyzed the effect of combining Bortezomib and Doramapimod on the colony formation ability of tumor cells. We found that the dual drug treatment significantly inhibited the colony formation of both MDA-MB-231 cells and A375 cells (Fig. 8H–K). Subsequently, we investigated the efficacy of Bortezomib and Doramapimod in A375 tumor xenografts and found that dual Bortezomib and Doramapimod treatment dramatically enhanced the antitumor activity compared to Bortezomib or Doramapimod alone (Fig. 8L–N). However, we also found that the strong antitumor activity of the combination strategy was also accompanied by toxic effects in terms of obvious weight loss (Fig. 8O), implying that further in-depth studies are warranted before clinical application. Collectively, these results suggest a new therapeutic intervention strategy for proteasome inhibitor application in tumor treatment.

## Discussion

Autophagy is a major protective mechanism for cells to remove aggregated misfolded proteins following cellular stresses, such as oxidative stress, heavy metal exposure and ER stress [6]. However, the role of autophagy in cellular stress induced by proteasome inhibitors is still unclear. Herein, we reported that autophagosome maturation was suppressed during the early stages of proteasome inhibition, which facilitated aggresome formation. Besides, SQSTM1 T269/S272 phosphorylation induced by proteasome inhibition could inhibit its autophagic receptor activity and promote aggresome formation (Fig. 8P). Moreover, our study suggests a novel therapeutic intervention for proteasome inhibitor-mediated tumor treatment.

Previous studies reported that proteasome activity impairment could activate the autophagic degradation of misfolded proteins in neuronal cells [51, 52]. However, in endothelial cells and multiple myeloma cells, inhibition of autophagy reversed proteasome inhibitor-induced cell death [53, 54]. In this study, we found that a low extent of proteasome inhibition suppressed autophagosome formation (Fig. 1). Despite prolonged proteasome inhibition activating the autophagy, this activation did not increase the autophagic degradation of polyubiquitinated proteins (Fig. 2). Consistently with these results, recent studies demonstrated that bortezomib-induced autophagy activation, which was occurred after 20-h exposure to a proteasome inhibitor, did not increase the degradation of ubiquitinated proteins in multiple myeloma and neuroblastoma cells [12]. In addition to proteasome inhibition, there are a variety of cellular stresses that could induce the aggregation of misfolded proteins, such as ER stress, ROS, etc., and it has been reported that these cellular stresses can activate the autophagic degradation of misfolded proteins [4, 55–57], suggesting that autophagic degradation of aggregated proteins is dependent on the type of cellular stress. Therefore, more detailed research is needed to reveal how autophagy differentiates the protein aggregates to regulate degradation in response to different cellular stress.

Upon proteasome inhibition, polyubiquitinated proteins are sequestered into aggregates and aggresomes are then formed at MTOC in a microtubule-dependent manner. Among the numerous protein factors that direct the aggresome formation, SQSTM1 reportedly plays a critical role in this process. Kehl et al. reported that the phosphorylation of SQSTM1, including at the T269/S272 site, mediated by TAK1, could inhibit the binding of SQSTM1 to LC3B and could dissociate the oligomeric SQSTM1 from autophagosomes [44]. In this study, we found that during proteasome inhibition, SQSTM1 T269/S272 phosphorylation also inhibited the presentation of SQSTM1 to the autophagosome (Fig. 6), which promoted their translocation into aggresomes, suggesting that phosphorylation of SQSTM1 T269/S272 might be a general mechanism that increases non-autophagic SQSTM1 level in response to cellular stress. Previous studies have revealed that the PB1 and UBA domains of SQSTM1 are critical regulators of aggresome formation by binding to polyubiquitinated proteins and promoting their aggregation [29, 58]. Here, we found that the phosphorylation failure of SQSTM1 T269/S272 did not affect the aggregation of ubiquitinated proteins (Fig. 4 and Supplementary Fig. S5), implying that phosphorylated SQSTM1 T269/S272 regulates aggresome formation may occur after protein aggregation. The transport of aggregated proteins is another important process after the protein aggregation in aggresome biogenesis. Since the HDAC6-Dynein motor complex plays a critical role in transporting micro-aggregates to the MTOC [8, 24, 25], we speculate that phosphorylated SQSTM1 (T269/S272) might promote the recognition of the HDAC6-Dynein motor complex. In addition, although proteasome inhibition decreases the level of autophagosome, the remaining autophagosomes are still able to induce the defective aggresome formation in the cells with failed SQSTM1 (T269/S272) phosphorylation, due to the defection could be rescued by inhibiting the autophagosome formation (Fig. 5). The evidence showed that excessive autophagosome presentation of misfolded proteins during proteasome inhibition could inhibit their transport to MTOC, thereby preventing aggresome formation. Future studies are warranted to address these possibilities.

What is puzzling is that although the failure of SQSTM1 T269/S272 phosphorylation could promote autophagosome localization, we have not observed the increase of polyubiquitinated proteins degradation by autophagy (Fig. 7). Zaarur et al. previously revealed that proteasome dysfunction causes lysosomal accumulation in the entrapment zone (E-zone) around the aggresome by inhibiting its random movement along the microtubule network [59]. Thus, these accumulated lysosomes might enhance the degradation of misfolded proteins transported into aggresome. Indeed, in the proteasome inhibitor washout experiment, we found that aggresomal ubiquitinated proteins in SQSTM1 (T269E/S272D)-expressing cells were more conducive to autophagic degradation, rather than the micro-aggregates in SQSTM1 (T269A/S272A)-expressing cells, although they mostly co-localized with autophagosomal marker (Fig. 6). One possibility is that the micro-aggregates-associated autophagosomes cannot efficiently fuse with lysosomes scattered in the cytosol during proteasome inhibition.

Mounting evidence suggests that aggresome formation of polyubiquitinated proteins could help cells deal with proteasome dysfunction through the effective sequestration of misfolded proteins to reduce proteotoxic stress [7–9, 12, 34, 48]. However, the role of autophagy in cell damage induced by proteasome suppression remains controversial. As shown in this study that raising autophagy cannot protect against cell damage caused by proteasome inhibition (Fig. 8G). Recent reports demonstrated that in Bortezomib-treated neuroblastoma, autophagy activation was later than widespread cell death [12]. Therefore, the activation of autophagy induced by proteasome inhibition might contribute more to cell damage than to the removal of aggregated proteins. However, it should be noted that the compensatory activation of autophagy following proteasome inhibition has been confirmed in other types of cells, such as glioblastoma [52], and hepatocytes [60], which might be because of the different autophagy systems in these cells. In tumorigenesis and cancer development, enhanced degradation of misfolded proteins is critical for cells to balance proteostasis due to excessive protein synthesis and numerous gene mutations [61, 62]. Thus, targeting of the proteasome has clinical significance in tumor therapy. Even though proteasome inhibitors have been approved to treat hematologic malignancy multiple myeloma (MM) for almost two decades, this approach was not extended to treat other malignancies. Our study found that Bortezomib, in combination with Doramapimod, exerted a better efficacy in treating A375 tumor xenografts (Fig. 8). This provides valuable insights for extending the application of proteasome inhibitors in tumors and solving the problem of drug resistance.

In summary, our study has revealed a molecular mechanism whereby autophagy disrupts the aggresome formation of misfolded proteins during proteasome inhibition. We identified that the phosphorylation of SQSTM1 (T269/S272) could suppress its autophagic receptor activity and promote the aggresome formation of ubiquitinated proteins during proteasome inhibition. Our results thus bring forward a novel therapeutic intervention strategy utilizing proteasome inhibitors to mediate tumor suppression.

## Supplementary information

Supplementary information

Uncroupped WB

AJ-checklist

## Data Availability

All data needed to evaluate the conclusions in the paper are present in the paper and/or the Supplementary Materials.
