# Supplementary material for: Autophagic sequestration of SQSTM1 disrupts the aggresome formation of ubiquitinated proteins during proteasome inhibition
Source: Cell Death Dis. 2022 Jul 15;13(7):615. doi: 10.1038/s41419-022-05061-8 (PMC9287315; doi:10.1038/s41419-022-05061-8)
Supplement: Supplementary file 1 — Supplementary information [file 41419_2022_5061_MOESM1_ESM.docx]

**Supplementary information**

**Autophagic sequestration of SQSTM1 disrupts the aggresome formation of ubiquitinated proteins during proteasome inhibition**

Chenliang Zhang^1^, Chen Huang^2^, Hongwei Xia^1^, Huanji Xu^2^, Qiulin Tang^1^, Feng Bi^1,2,*^

1 Laboratory of Molecular Targeted Therapy in Oncology, West China Hospital of Sichuan University, Chengdu, China

2 Department of Medical Oncology, Cancer Center, West China Hospital of Sichuan University, Chengdu, China

***Correspondence to:** Feng Bi, Email: [bifeng@scu.edu.cn](mailto:bifeng@scu.edu.cn)


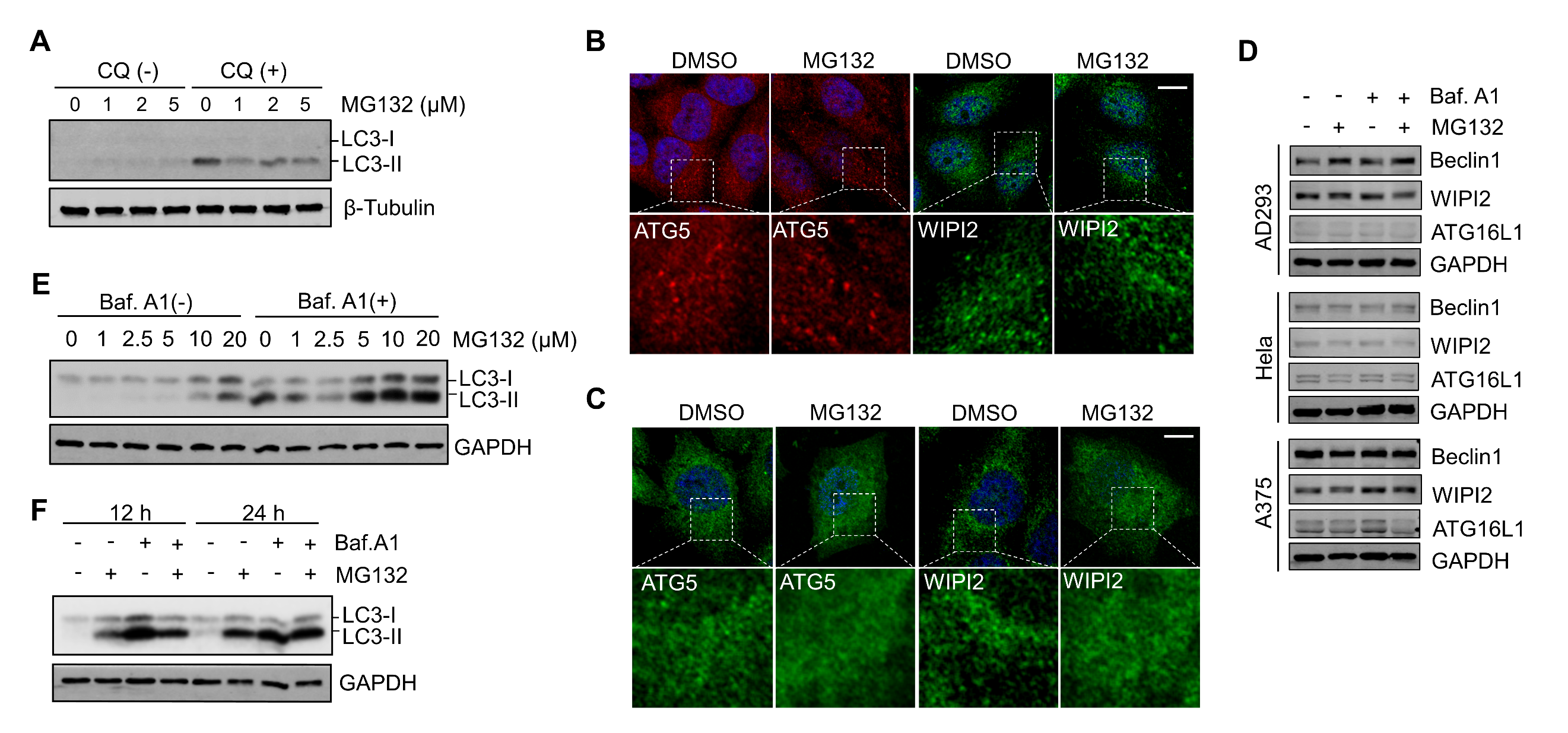


**Figure S1 Suppressing autophagy is the early response of cells to proteasome inhibition.**

**(A)** Hela cells treated with 2 μM MG132 with or without chloroquine (25 μM) for 14 h and then the whole cell lysates were subjected to western blot analysis with indicated antibodies.

**(B and C)** Hela (A) and A375 (B) cells were treated with DMSO or MG132 (2 μM) for 14 h, then fixed and immunostained with ATG5 or WIPI2 antibodies. Nuclei were stained with DAPI (blue). Scale bar: 10 μm. **(D)** AD293, Hela and A375 cells were treated with DMSO (control), MG132 (2 μM), and Bafilomycin A1 (25 nM), alone or in combination for 14 h. The whole cell lysates were subjected to western blot analysis with indicated antibodies. **(E)** AD293 cells were treated with MG132 at indicated concentrations with or without Bafilomycin A1 (25 nM) for 14 h, and then the whole cell lysates were subjected to western blot analysis with indicated antibodies. **(F)** AD293 cells were treated with 2 μM MG132 with or without Bafilomycin A1 (25 nM) for indicated time, and then the whole cell lysates were subjected to western blot analysis with indicated antibodies.


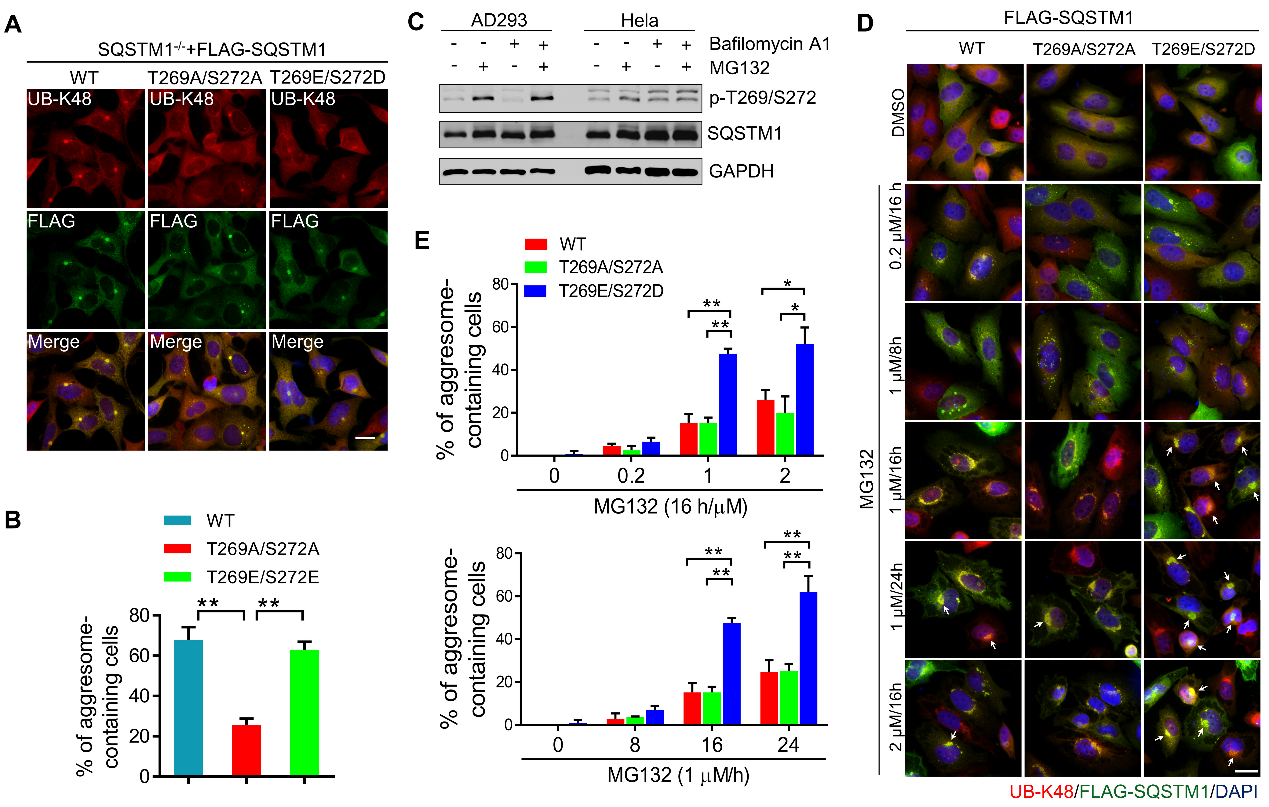


**Figure S2 Phosphorylation of SQSTM1 T269/S272 promotes proteasome inhibition-induced aggresome formation.**

**(A)** SQSTM1 knockout AD293 cells stably re-expressing FLAG-SQSTM1(WT or mutants) were treated with MG132 (2 μM) for 14 h. The aggresome formation was analyzed by immunostaining with anti-UB-K48 (Red) and anti-FLAG (Green) antibodies. Nuclei were stained with DAPI (blue). Scale bar: 20 μm. **(B)** Quantitative analysis of results in (A). **(C)** AD293 and Hela cells were treated with DMSO (control), or MG132 (2 μM), or Bafilomycin A1 (25 nM), or MG132 (2 μM)/ Bafilomycin A1 (25 nM) for 14 h. Next, the whole cell lysates were subjected to western blot analysis with indicated antibodies. **(D)** Hela cells stably expressing FLAG-SQSTM1 (WT or mutants) were treated with MG132 at indicated conditions. The aggresome formation of polyubiquitinated proteins was analyzed by immunostaining with anti-UB-K48 (Red) and anti-FLAG (Green) antibodies. Nuclei were stained with DAPI (blue). Scale bar: 20 μm. **(E)** Quantitative analysis of results in (D). For B and E, data are mean ± SEM. of 3 independent experiments. At least 40 cells were randomly selected to score, *=*p*<0.05, **=*p*<0.01.


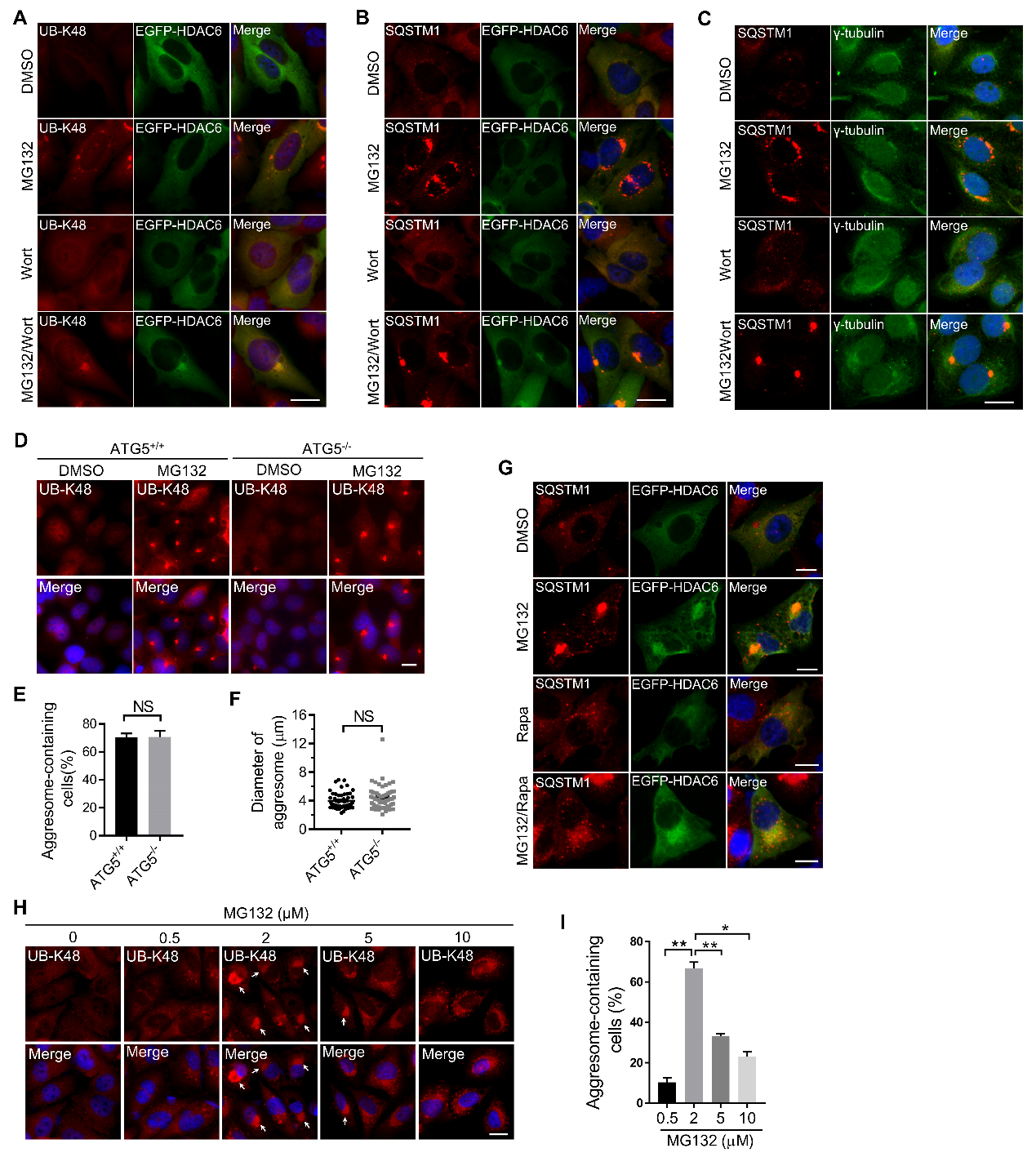


**Figure S3 Blocking autophagy promotes proteasome inhibitor-induced aggresome formation.**

**(A and B)** Hela cells were transfected with plasmids expressing EGFP-HDAC6 for 24 h, and then treated with DMSO (control), MG132 (1 μM), and Wortmannin (5 μM), alone or in combination for 14 h. The aggresome formation was analyzed by immunostaining with anti-UB-K48, anti-SQSTM1 (Green) antibodies. Nuclei were stained with DAPI (blue). Scale bar: 20 μm. **(C)** Hela cells were treated with DMSO (control), MG132 (1 μM), and Wortmannin (5 μM), alone or in combination for 14 h. The cells were then fixed and immunostained with anti-SQSTM1 and anti-γ-tubulin. Nuclei were stained with DAPI (blue). Scale bar: 20 μm. **(D)** Wild type (ATG5^+/+^) or ATG5 knockout (ATG5^-/-^) AD293 cells were treated with DMSO (control), or MG132 (2 μM) for 14 h. The aggresome formation of polyubiquitinated proteins was analyzed by immunostaining with anti-UB-K48 antibody. Nuclei were stained with DAPI (blue). Scale bar: 20 μm. **(E and F)** Quantitative analysis of aggresome formation and the size of aggresome showed in (D). **(G)** A375 cells were transfected with plasmids expressing EGFP-HDAC6 for 24 h, and then pretreated with or without Rapamycin (2 μM) for 10 h following DMSO (control) or MG132 (2 μM) treatment for 14 h. The cells were then fixed and immunostained with anti-SQSTM1 antibodies. Nuclei were stained with DAPI (blue). Scale bar: 20 μm. **(H)** A375 cells were treated with MG132 at indicated concentrations for 14 h. The aggresome formation of polyubiquitinated proteins was analyzed by immunostaining with anti-UB-K48 (Red) antibody. Nuclei were stained with DAPI (blue). Scale bar: 20 μm. **(I)** Quantitative analysis of results in (H). For E, F and I, data are mean ± SEM. of 3 independent experiments. At least 40 cells were randomly selected to score, *=*p*<0.05, **=*p*<0.01, NS=no significance.


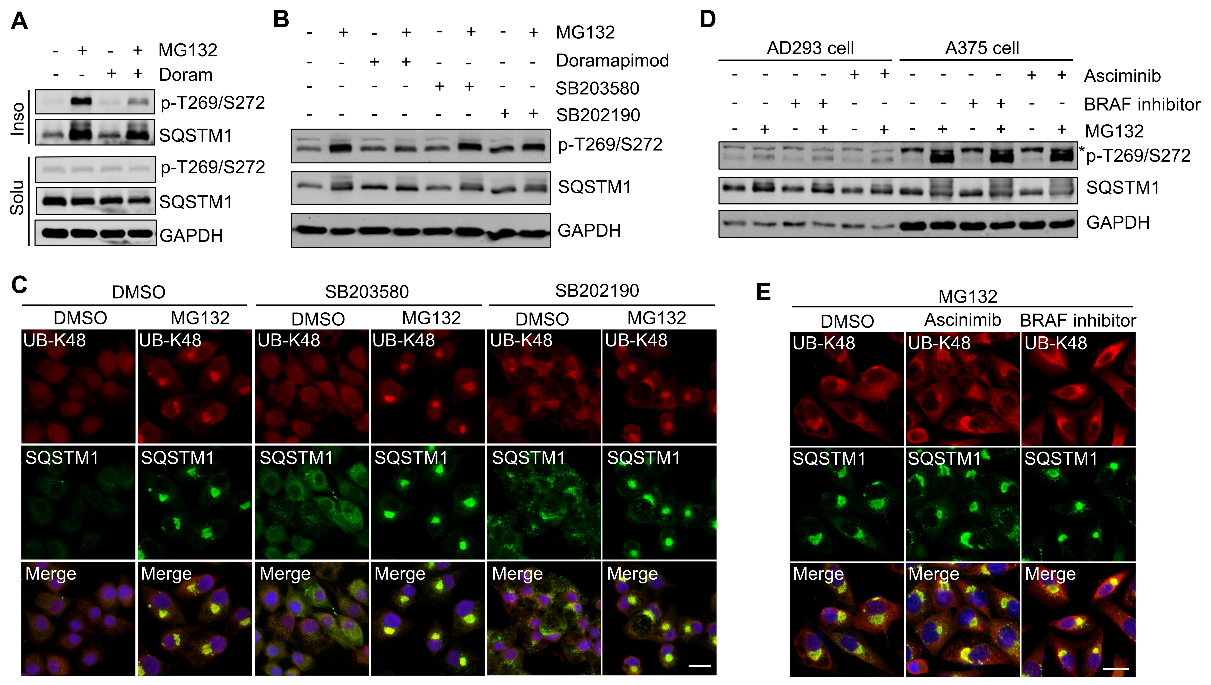


**Figure S4 Doramapimod reduces the phosphorylation of SQSTM1 (T269/S272) by inhibiting p38γ/p38δ kinase activity.**

**(A)** AD293 cells were treated with DMSO (control), MG132 (2 μM), and Doramapimod (50 μM), alone or in combination for 14 h. Next, whole cell extracts were separated into NP40-soluble and -insoluble fractions and subjected to western blot analysis with indicated antibodies. **(B)** A375 cells were treated with indicated inhibitors (MG132 (2 μM), Doramapimod (50 μM), SB203580 (20 μM), SB202190 (20 μM)) for 14 h. The whole cell lysates were subjected to western blot analysis with indicated antibodies. **(C)** A375 cells were treated with indicated inhibitors (MG132 (2 μM), Doramapimod (50 μM), SB203580 (20 μM), SB202190 (20 μM)) for 14 h. The aggresome formation was analyzed by immunostaining with anti-UB-K48 and anti-SQSTM1 antibodies. Nuclei were stained with DAPI (blue). Scale bar: 20 μm. **(D)** A375 cells were treated with indicated inhibitors (MG132 (2 μM), Asciminib (10 μM), BRAF inhibitor (5 μM)) for 14 h. The whole cell lysates were subjected to western blot analysis with indicated antibodies. **(E)** A375 cells were treated with indicated inhibitors (MG132 (2 μM), Asciminib (10 μM), BRAF inhibitor (5 μM)) for 14 h. The aggresome formation was analyzed by immunostaining with anti-UB-K48 and anti-SQSTM1 antibodies. Nuclei were stained with DAPI (blue). Scale bar: 20 μm.


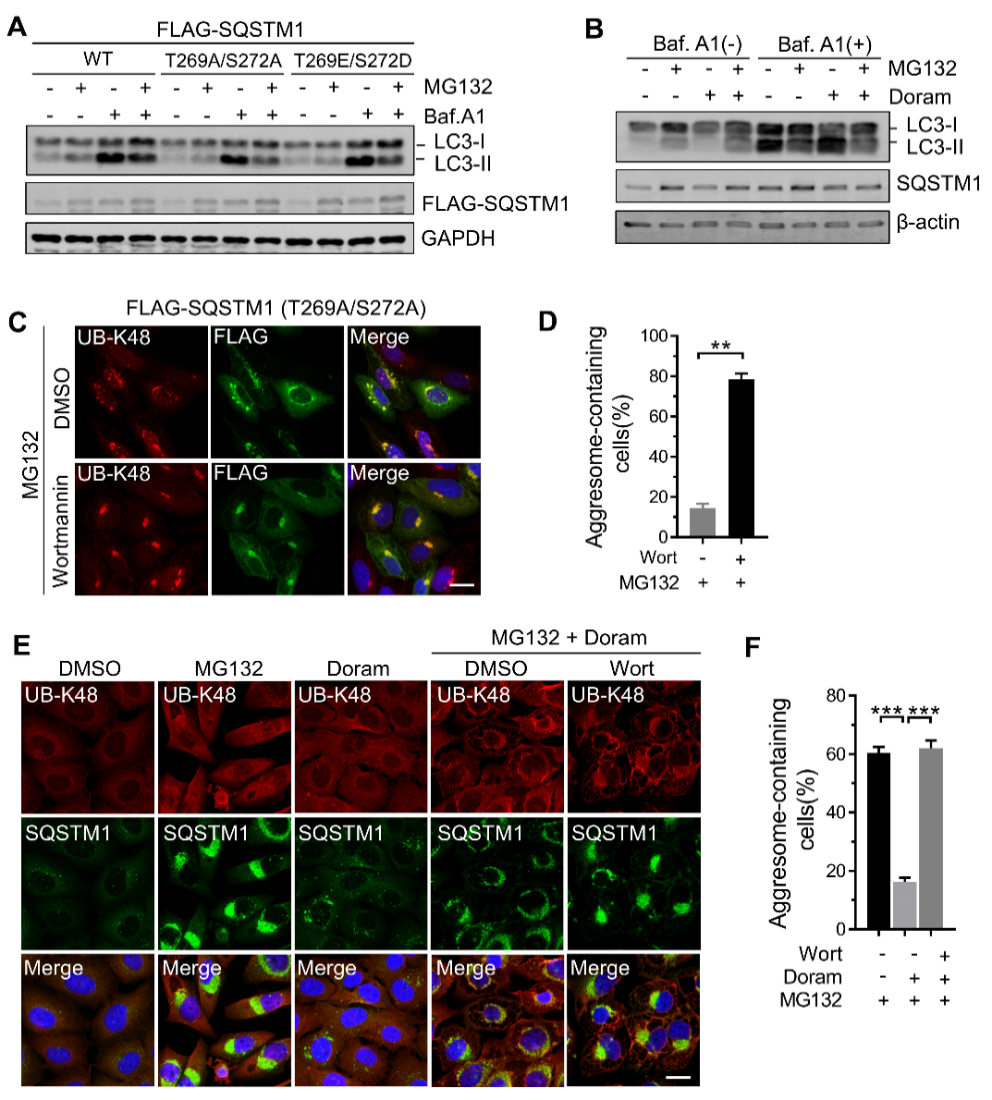
**Figure S5** **Blocking autophagy rescues the defective aggresome formation caused by failed phosphorylation of SQSTM1 (T269/S272)**

**(A)** Hela cells stably expressing FLAG-tagged SQSTM1 (wild-type (WT) or mutants) were treated with DMSO (control), MG132 (2 μM), and Bafilomycin A1 (25 nM), alone or in combination for 14 h. Next, the whole cell lysates were subjected to western blot analysis with indicated antibodies. **(B)** AD293 cells were treated with indicated inhibitors (MG132 (2 μM), Doramapimod (50 μM), Bafilomycin A1 (25 nM)) for 14 h. The whole cell lysates were subjected to western blot analysis with indicated antibodies. **(C)** Hela cells stably expressing FLAG-tagged SQSTM1 (T269A/S272A) were treated with MG132 (2 μM), or MG132 (2 μM)/ Wortmannin (5 μM) for 14 h. The aggresome formation was analyzed by immunostaining with anti-UB-K48 and anti-SQSTM1 antibodies. Nuclei were stained with DAPI (blue). Scale bar: 20 μm. **(D)** Quantitative analysis of results in (C). **(E)** MDA-MB-231 cells were treated with indicated inhibitors (MG132 (2 μM), Doramapimod (50 μM), Wortmannin (5 μM)) for 14 h, and then were fixed and immunostained with anti-UB-K48 and anti-SQSTM1 antibodies. Nuclei were stained with DAPI (blue). Scale bar: 20 μm. **(F)** Quantitative analysis of results in (E). For D and F, at least 50 cells were randomly selected from each group to score for aggresomes. Data are mean ± SEM. of three independent experiments. *=*p*<0.05, **=*p*<0.01, ***=*p*<0.001.


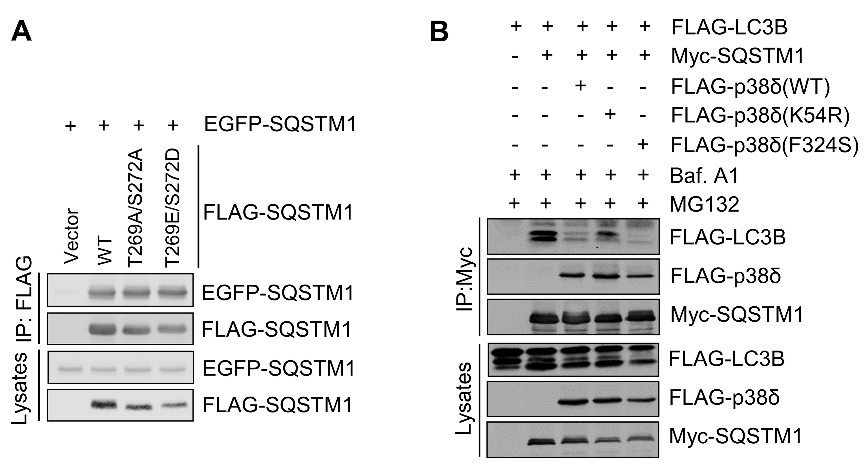


**Figure S6 SQSTM1(T269/S272) phosphorylation inhibits its autophagic receptor activity upon proteasome inhibition.**

**(A)** AD293 cells were transfected with indicated plasmids for 24 h, and then treated with MG132 (10 μM) for 5 h. The cells were lysed and immunoprecipitated with anti-FLAG antibody. Co-immunoprecipitated proteins were detected by western blot analysis using indicated antibodies. **(B)** AD293 cells were transfected with indicated plasmids for 24 h, and then treated with MG132 (10 μM) and Bafilomycin A1 (25 nM) for 5 h. Cell lysates were immunoprecipitated with anti-Myc antibodies. Co-immunoprecipitated proteins were detected by western blot analysis using indicated antibodies.


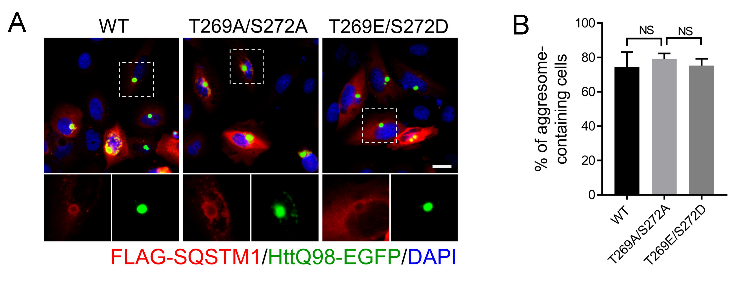


**Figure S7 Non-phosphorylation of SQSTM1 (T269/S272) does not affect the proteasome inhibition-independent aggresome formation.**

**(A)** Hela cells stably expressing FLAG-SQSTM1 (WT or mutants) were transfected with the plasmids expressing EGFP tagged HttQ98 for 24 h, and then were fixed and stained with FLAG antibody. Nuclei were stained with DAPI (blue). Scar bar: 20 μm. **(B)** Quantitative analysis of results in (B). At least 50 cells were randomly selected from each group to score for aggresomes. Data are mean ± SEM. of three independent experiments. NS=not significant.

| **Table S1. Sequences of the primers used for ORF amplification** | | |
| --- | --- | --- |
| **DNA Construct** | **PCRed DNA Fragment** | **Primer Name and Sequence (5'-3')** |
| LC3B | LC3B | LC3B-f：gatcggatccgccaccatgccgtcggagaagaccttcaagc |
|  |  | LC3B-r：gatctctagacactgacaatttcatcccgaacgtctcc |
| HDAC6 | HDAC6 | HDAC6-f：gatcaagcttatgacctcaaccggccaggattccac |
|  |  | HDAC6-r：gatctctagattagtgtgggtggggcatatcctcccc |
| SQSTM1 (WT or mutants) | SQSTM1 | SQSTM1-f：gatcgaattcatggcgtcgctcaccgtgaaggcctac |
|  |  | SQSTM1-r：gatcctcgagtcacaacggcgggggatgctttgaatac |

**Table S2 Antibody information**

| **Antibody** | **Company** | **Cat #** | **Dilution** |
| --- | --- | --- | --- |
| UB-K48 | Millipore | 05-1307 | WB: 1:1000 IF: 1:300 |
| UB-K63 | Abcam | ab179434 | WB: 1:1000  WB: 1:200 |
| ubiquitin | Abcam | ab134953 | WB: 1:1000 |
| Phospho-SQSTM1 (Thr269/Ser272) | Phosphosolutions | P196-269 | WB: 1:1000 |
| SQSTM1 | Santa Cruz Biotechnology | sc-28359 | WB: 1:2000 IF: 1:300 |
| ATG5 | Cell Signaling Technology | 12994 | WB: 1:1000  IF: 1:200 |
| LC3B | Cell Signaling Technology | 3868 | WB: 1:1000  WB: 1:200 |
| WIPI2 | Abcam | ab105459 | WB: 1:1000  WB: 1:200 |
| Beclin1 | Proteintech | 11306-1-AP | WB: 1:1000 |
| ATG16L1 | Abcam | ab187671 | WB: 1:500 |
| γ-tubulin | Novus | NBP2-43585 | WB: 1:200 |
| GFP | Rockland | 600-101-215 | WB: 1:2000 |
| FLAG tag | Prospec | ANT-146-b | WB: 1:2000 IF: 1:200 |
| Myc Tag | Biolegend | MMS-150R | WB: 1:2000 |
| GAPDH | Zen Bioscience | 200306 | WB: 1:5000 |
| β-actin | Zen Bioscience | 200068-6D7 | WB: 1:5000 |
