# Supplementary material for: Autophagic sequestration of SQSTM1 disrupts the aggresome formation of ubiquitinated proteins during proteasome inhibition
Source: Cell Death Dis. 2022 Jul 15;13(7):615. doi: 10.1038/s41419-022-05061-8 (PMC9287315; doi:10.1038/s41419-022-05061-8)

Fig 1A

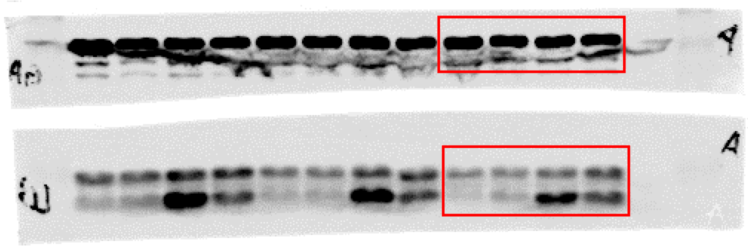

Fig 1B

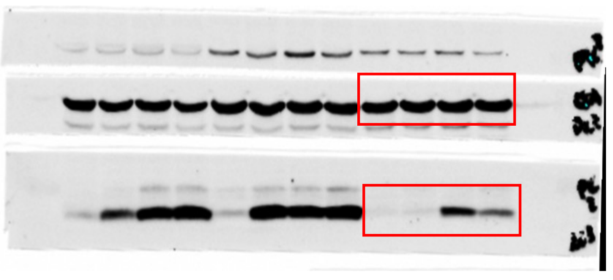

Fig 1C

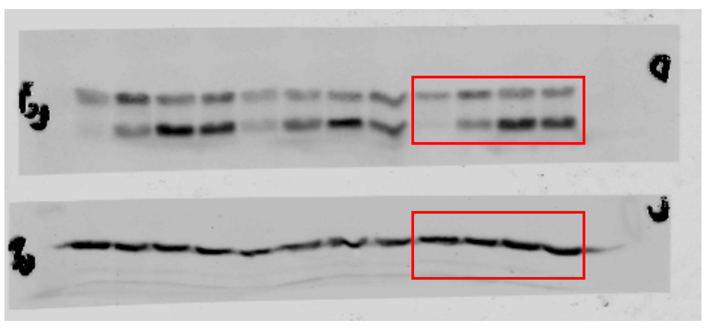

Fig 1I

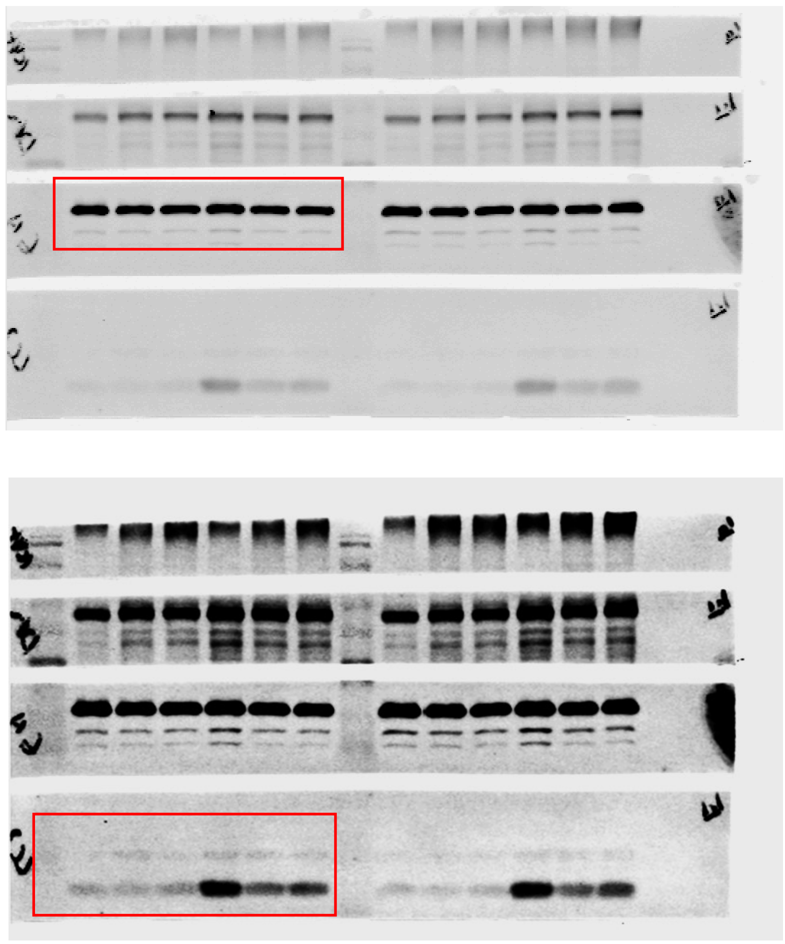

Fig 1K

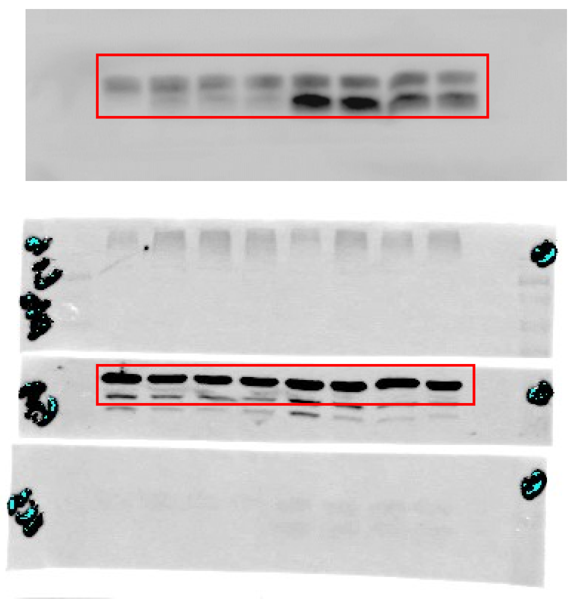

Fig 2A

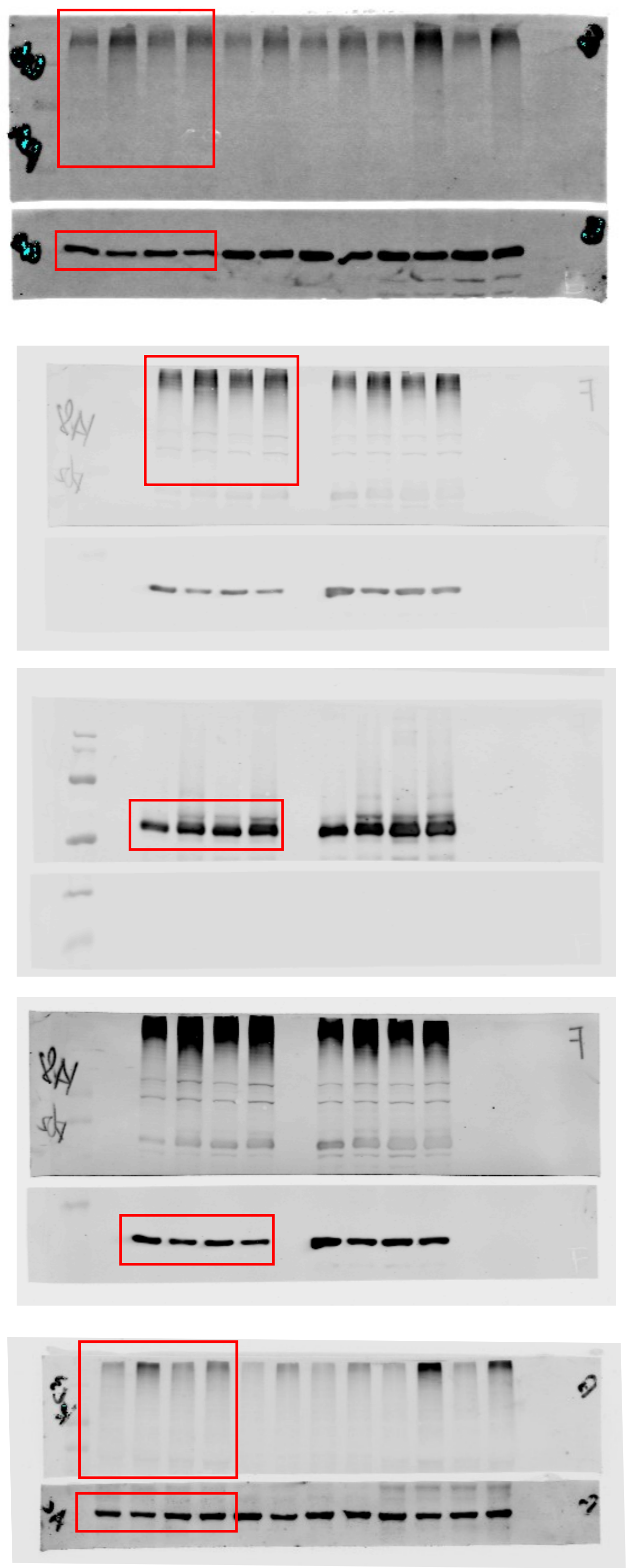

Fig 2C

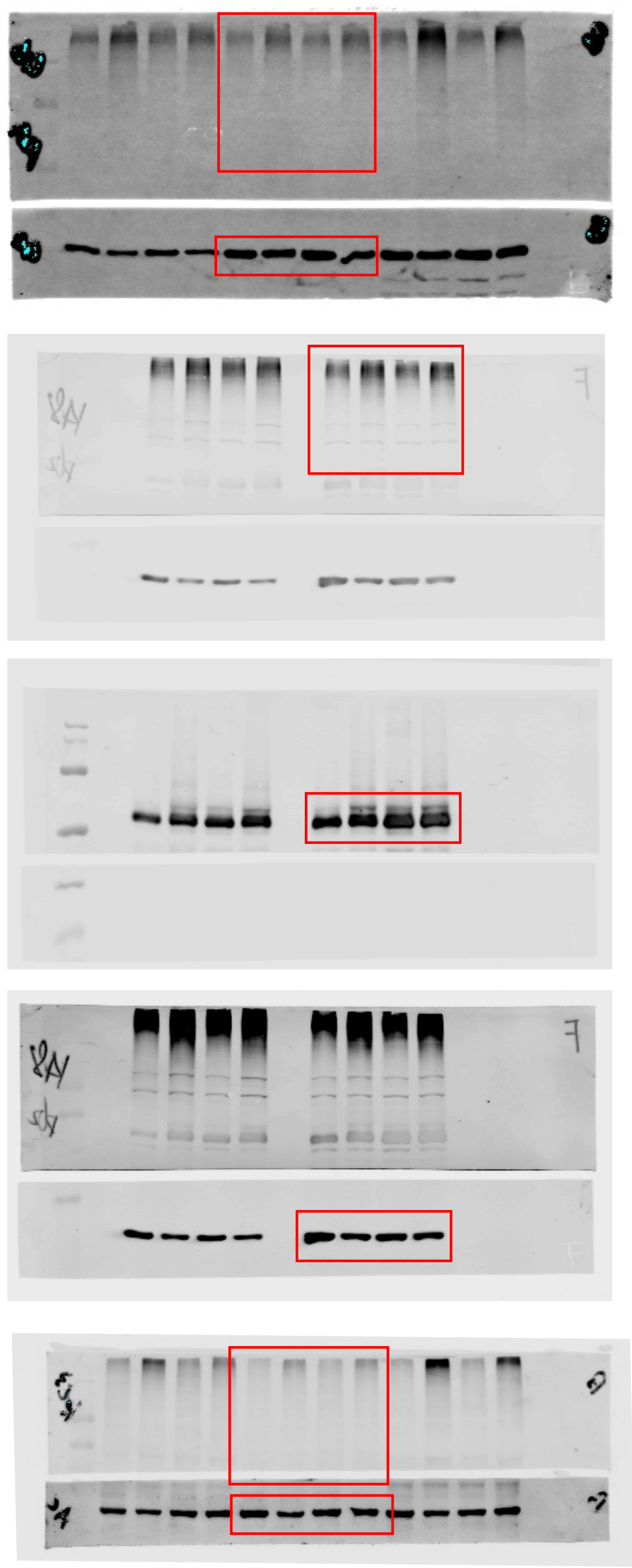

Fig 2D

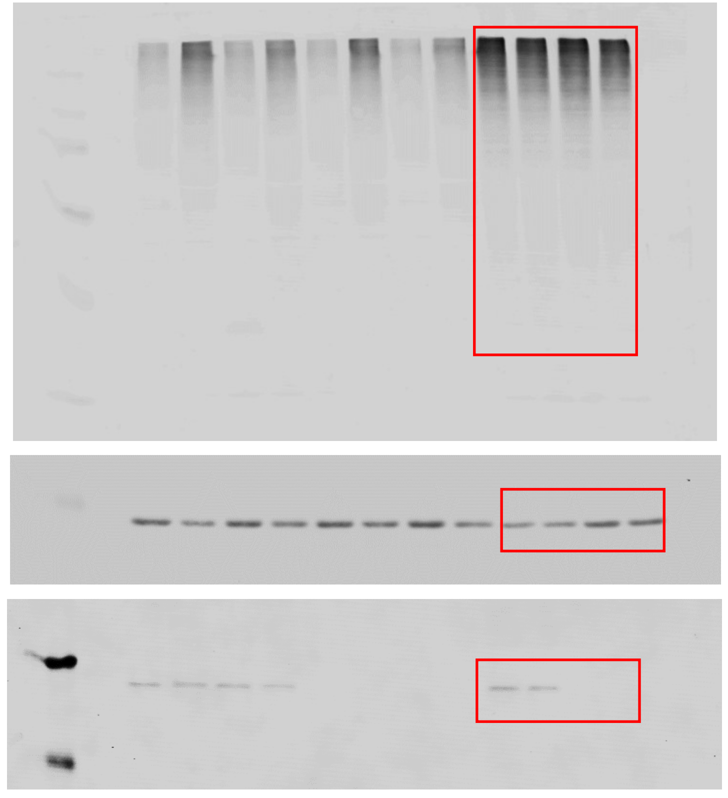

Fig 2B

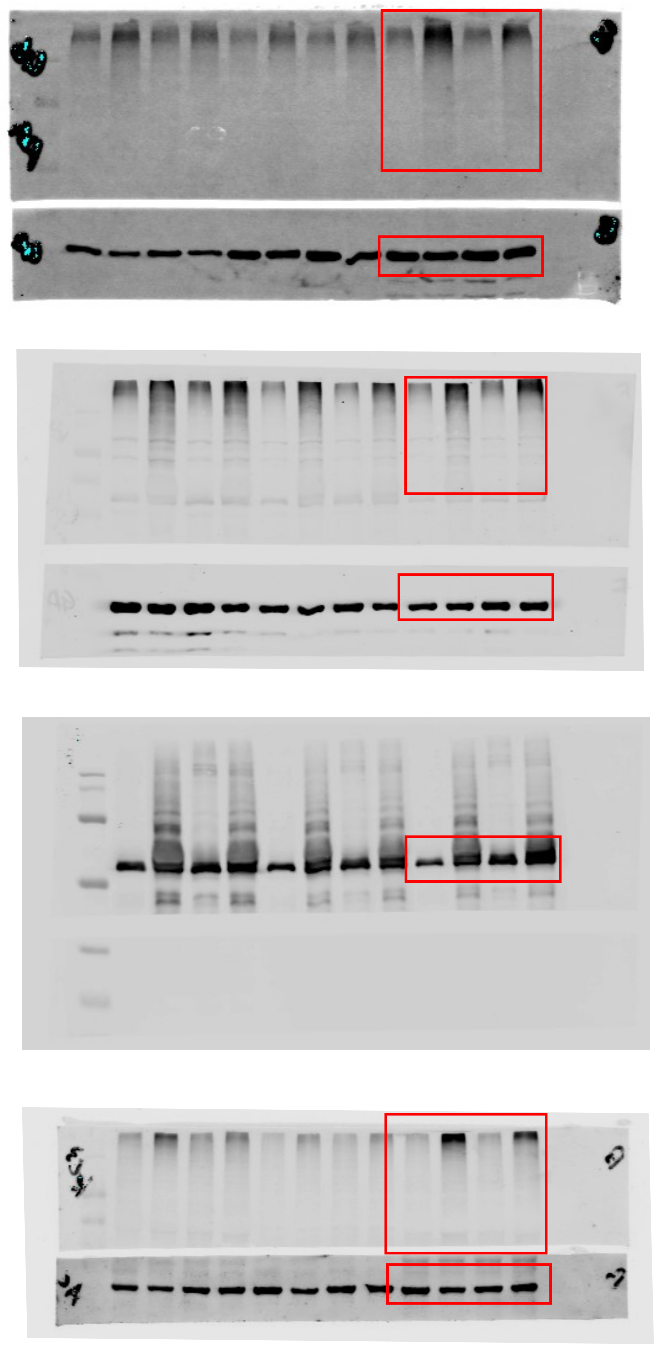

Fig 2E

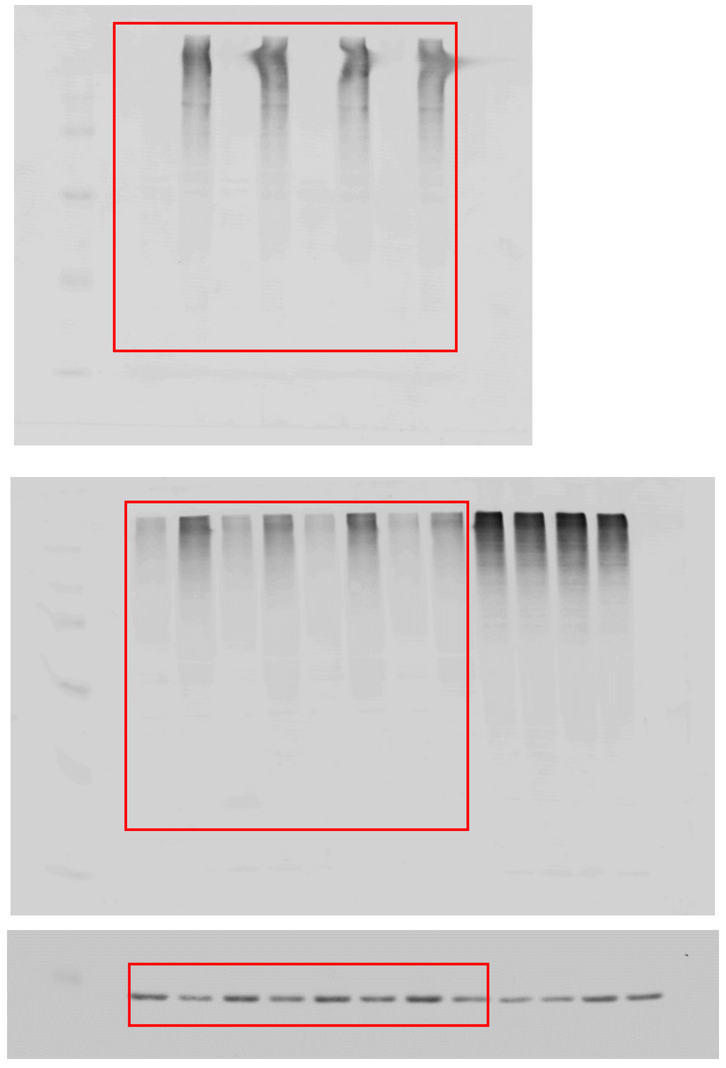

Fig 2F

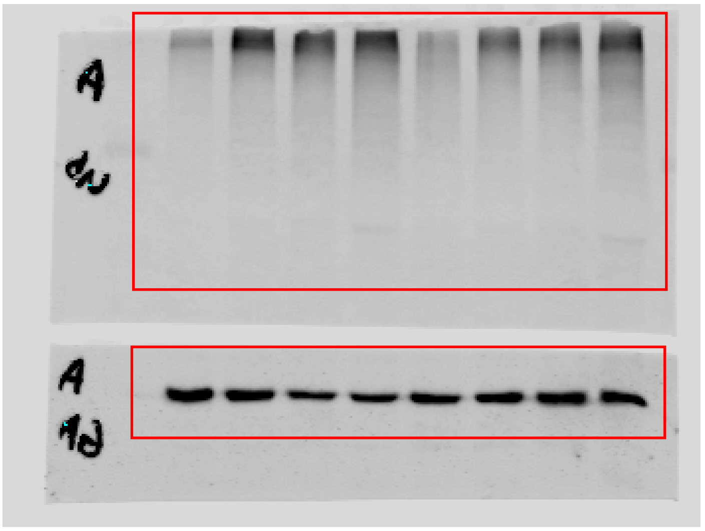

Fig 2G

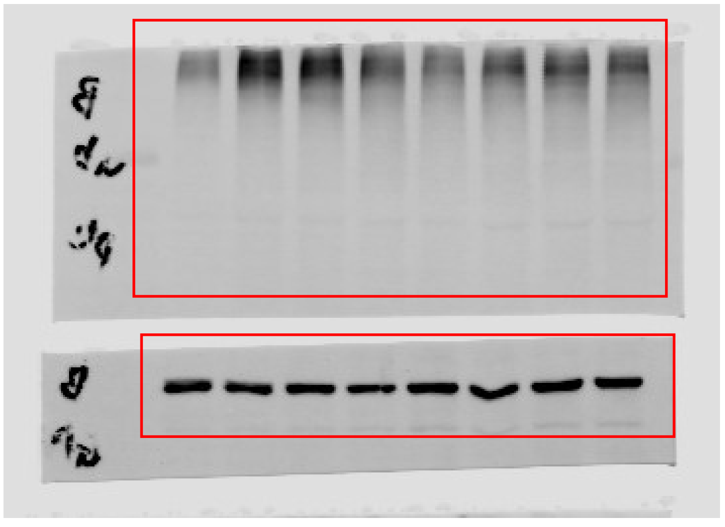

Fig 2H

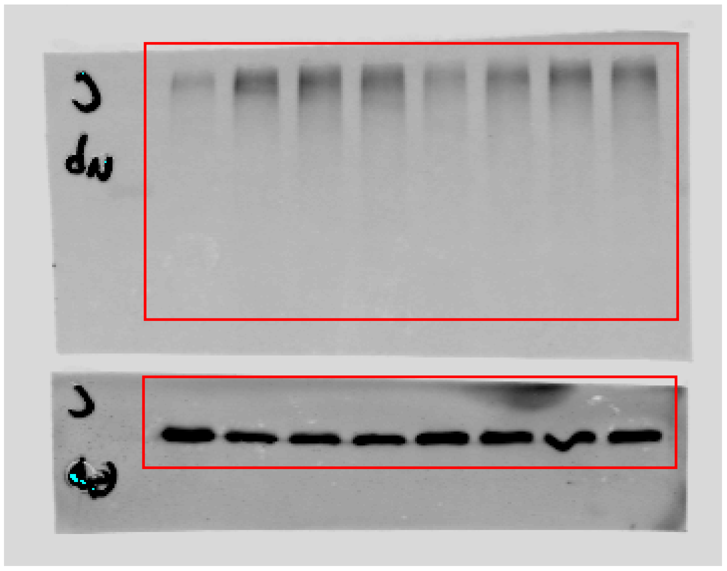

Fig 2I

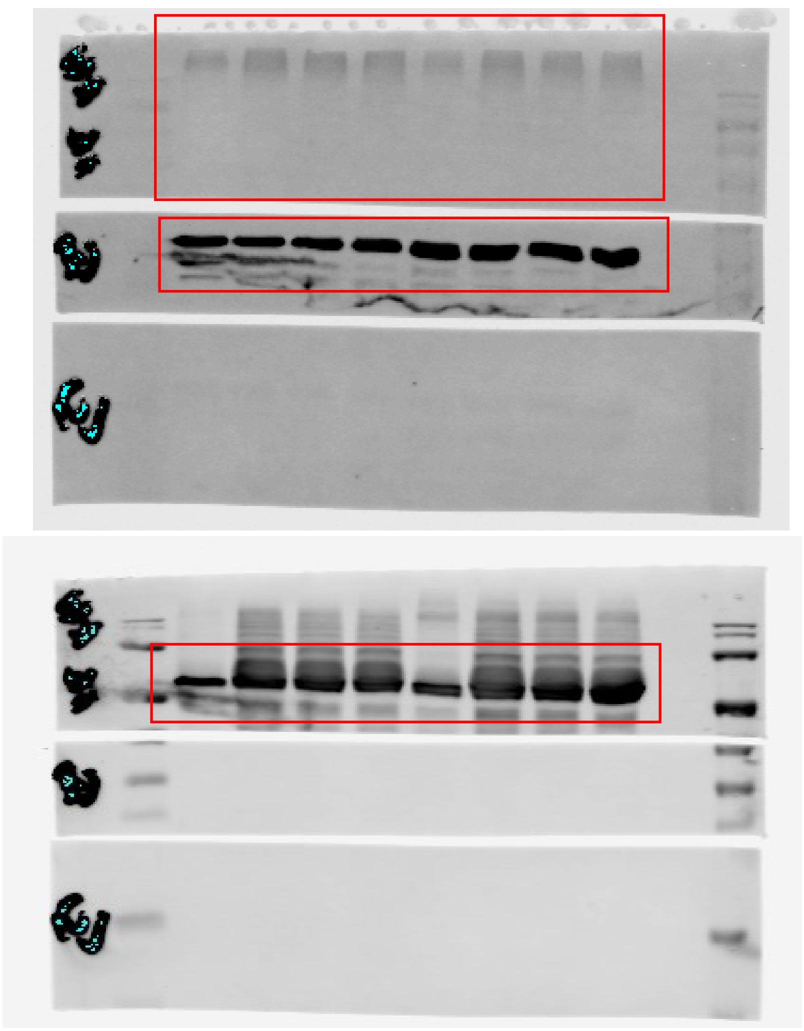

Fig 2J

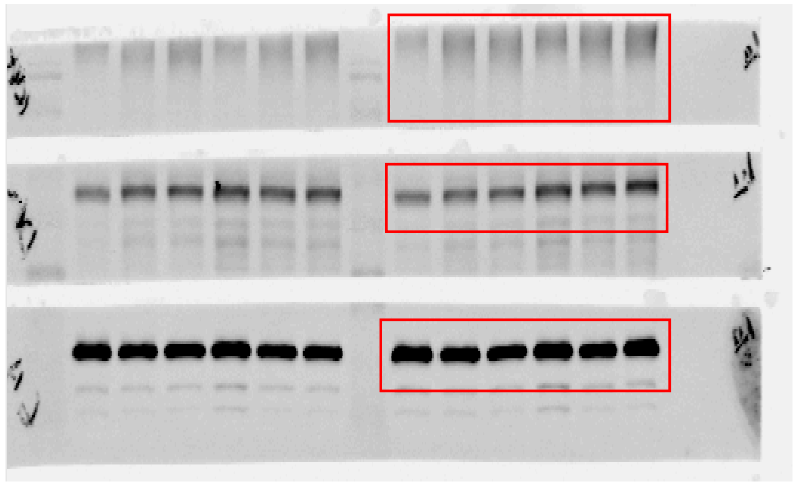

Fig 3A

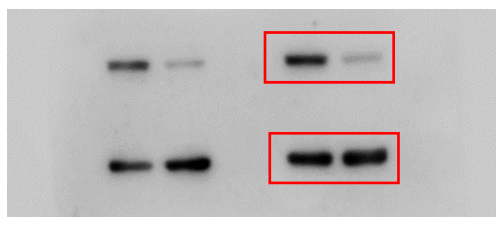

Fig 4A

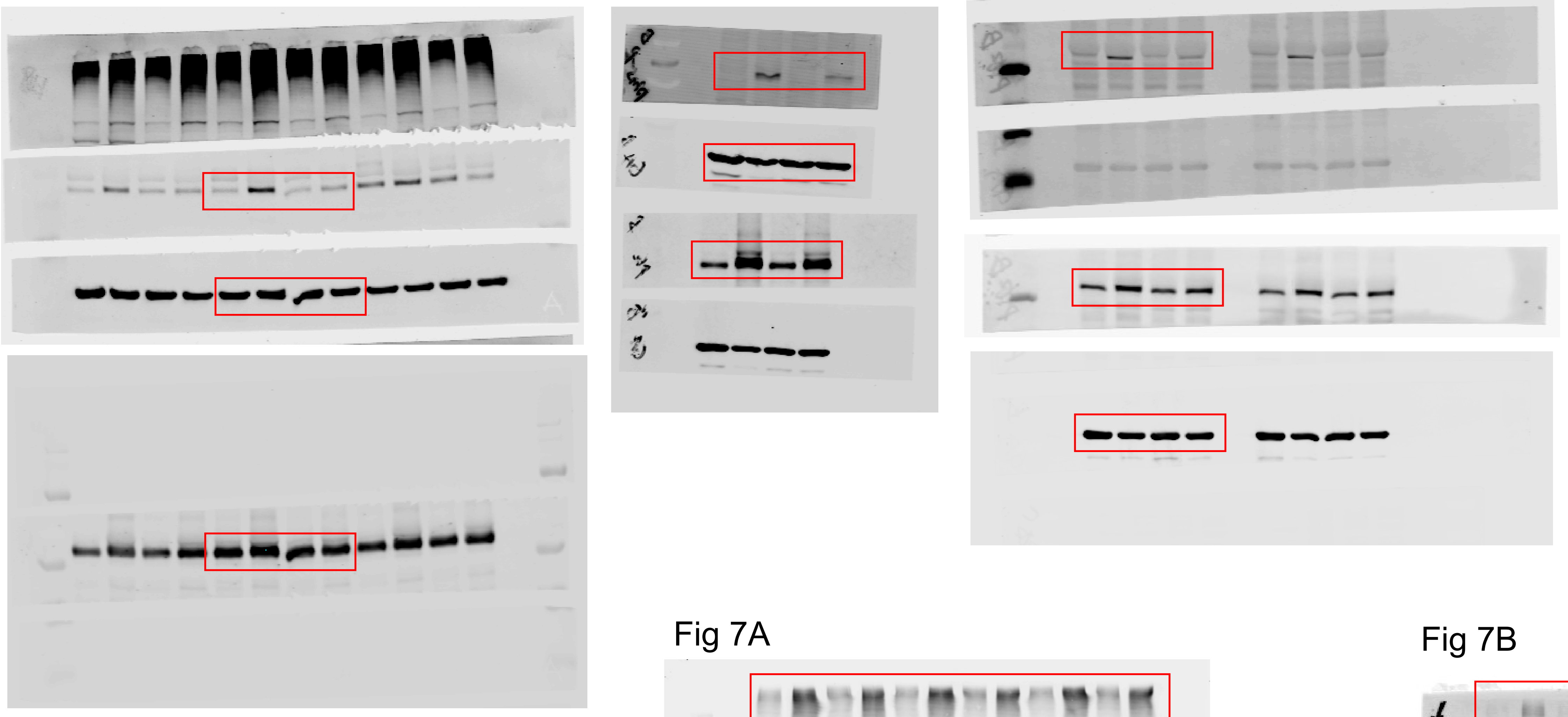

Fig 4C

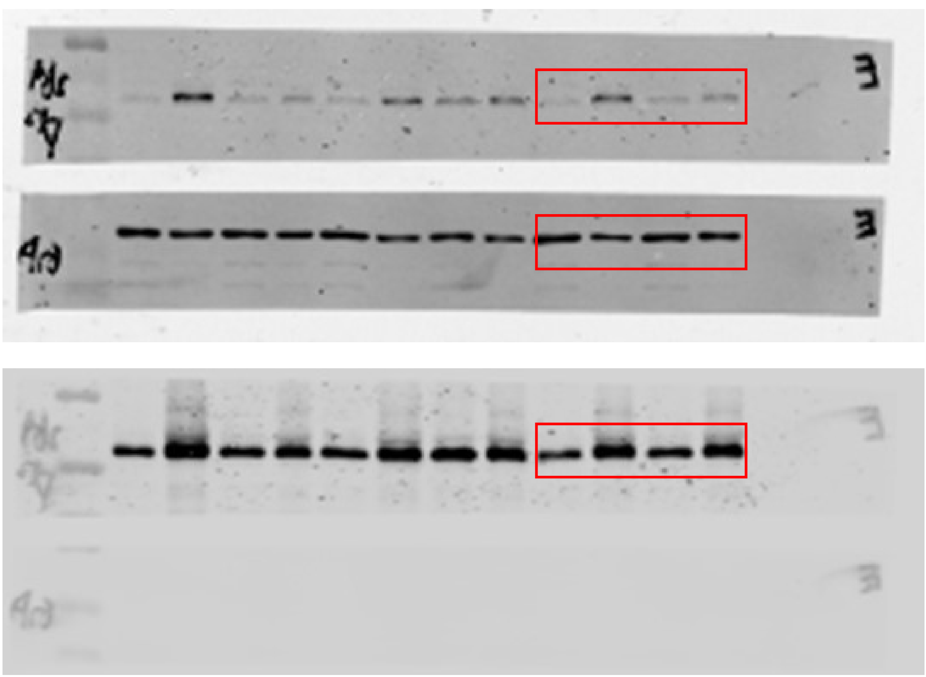

Fig 7A

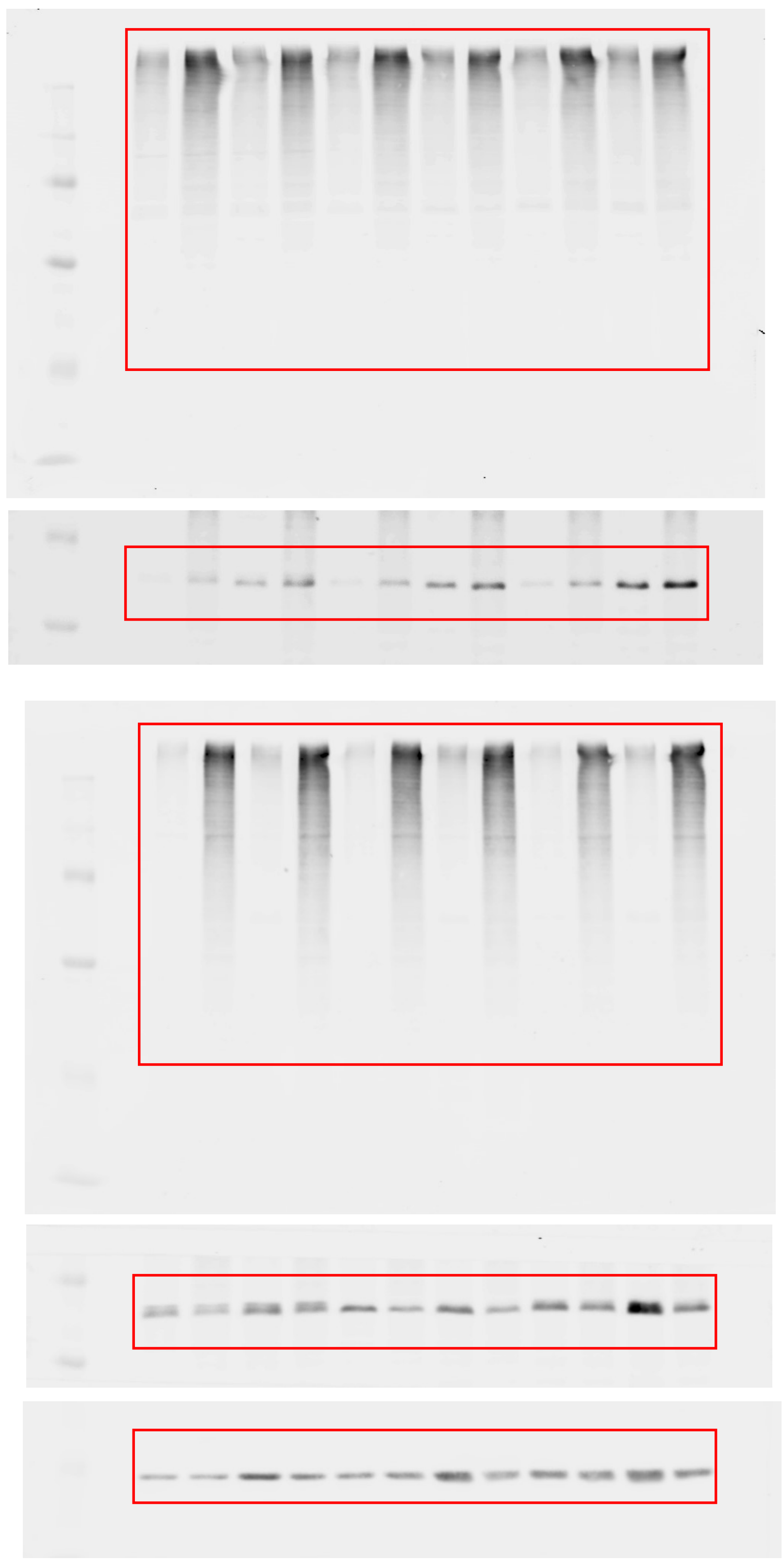

Fig 7B

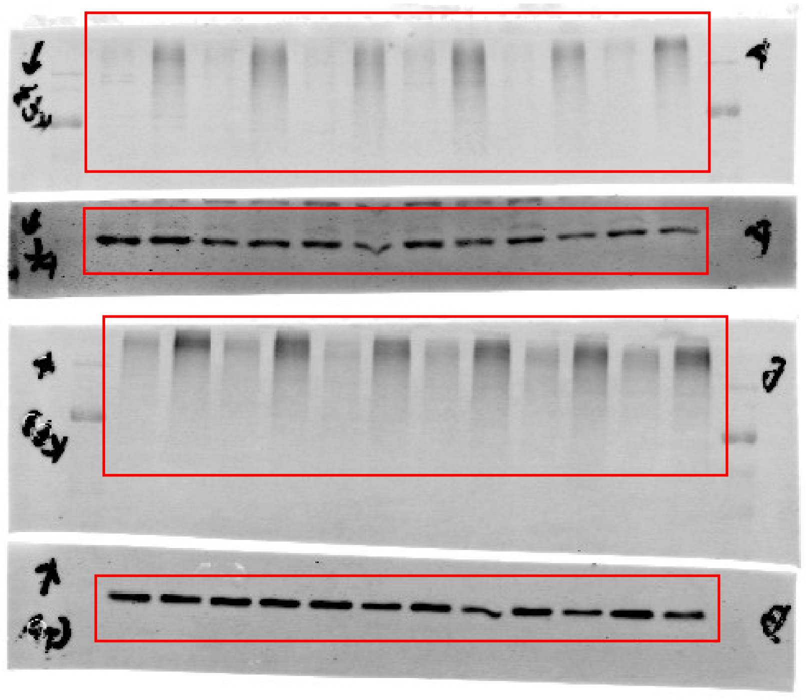

Fig 6A

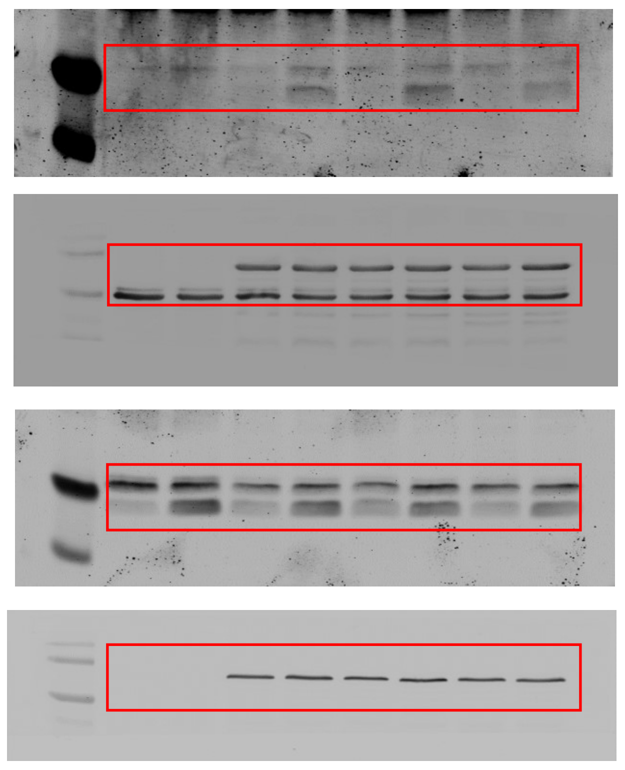

Fig 7C

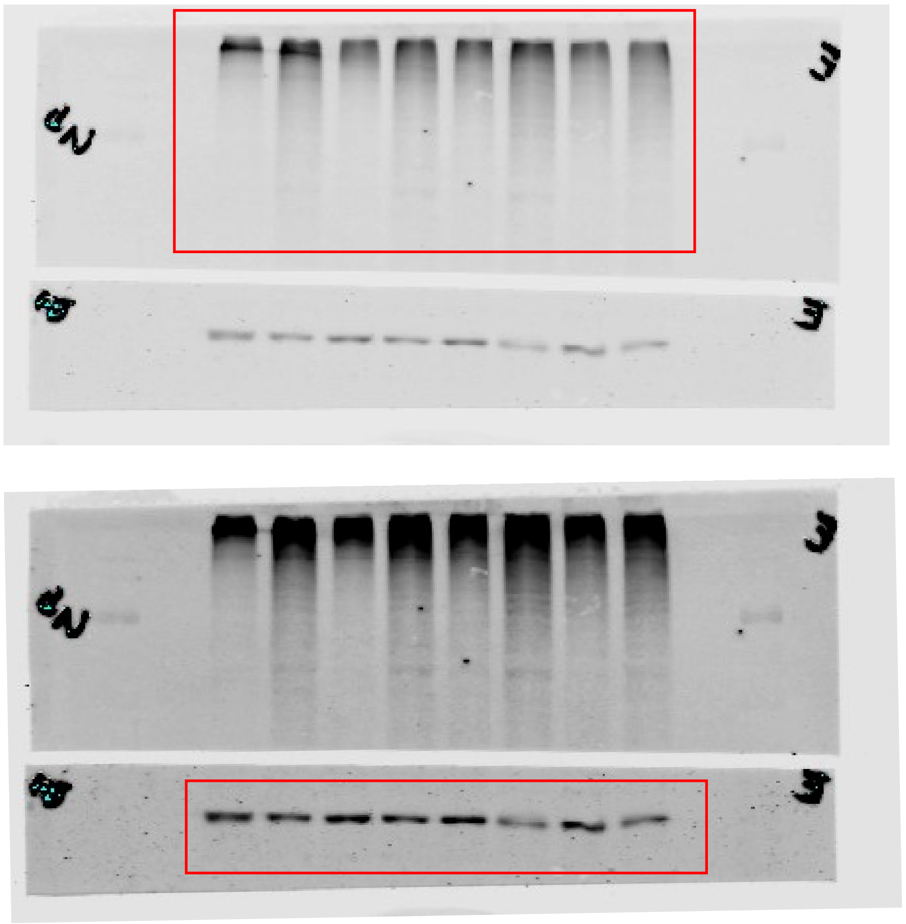

Fig 7D

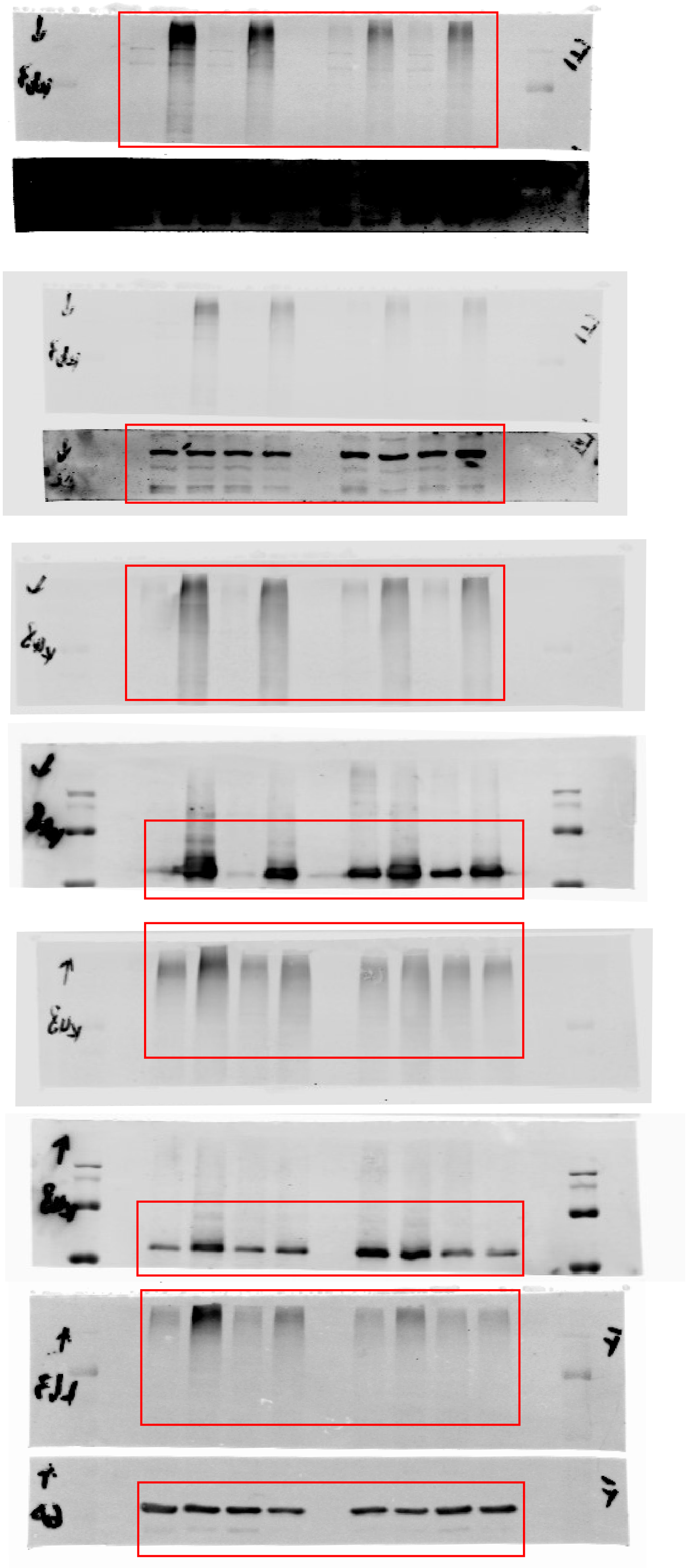

Fig 7G

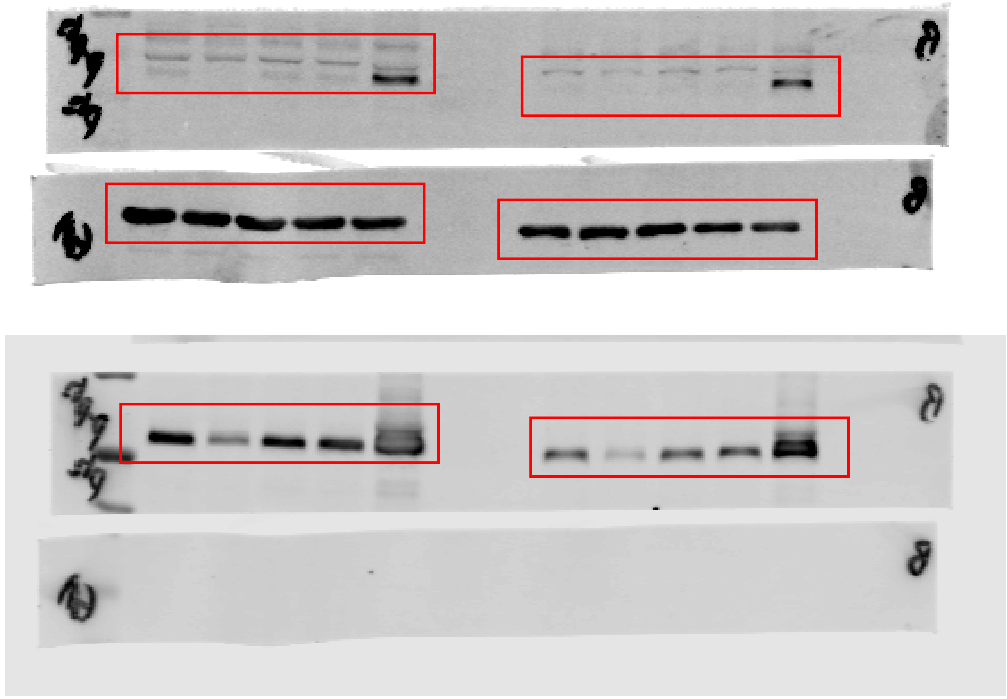

Fig 7H

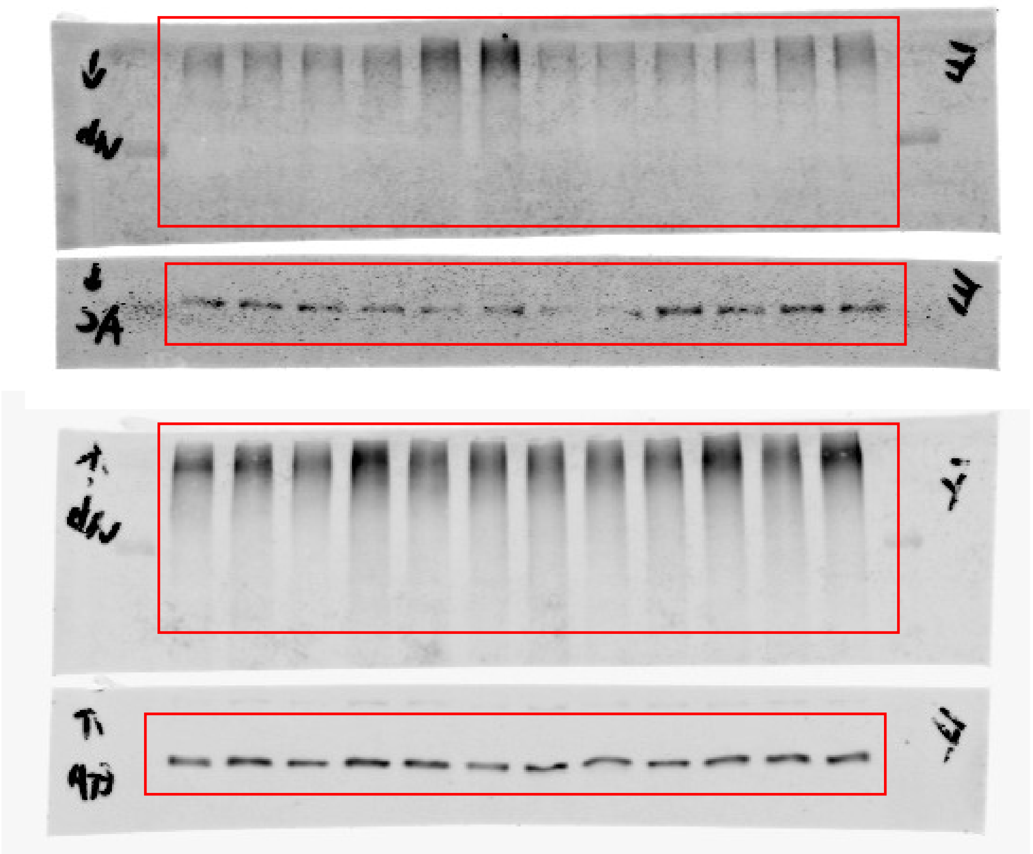

Fig S1A

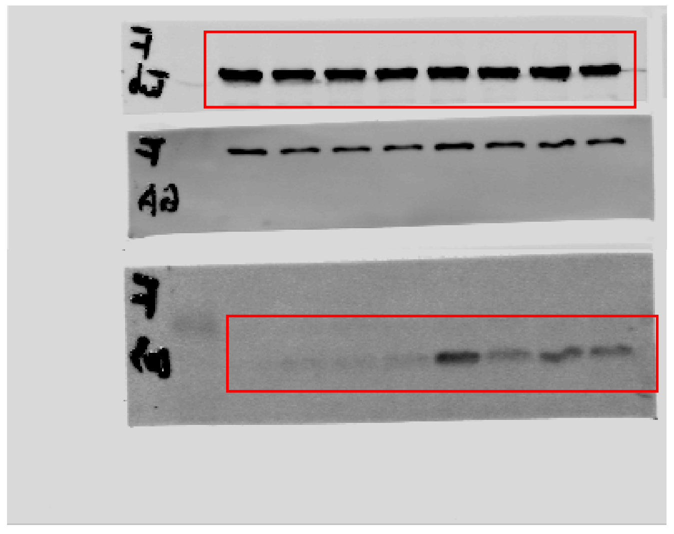

Fig S1D

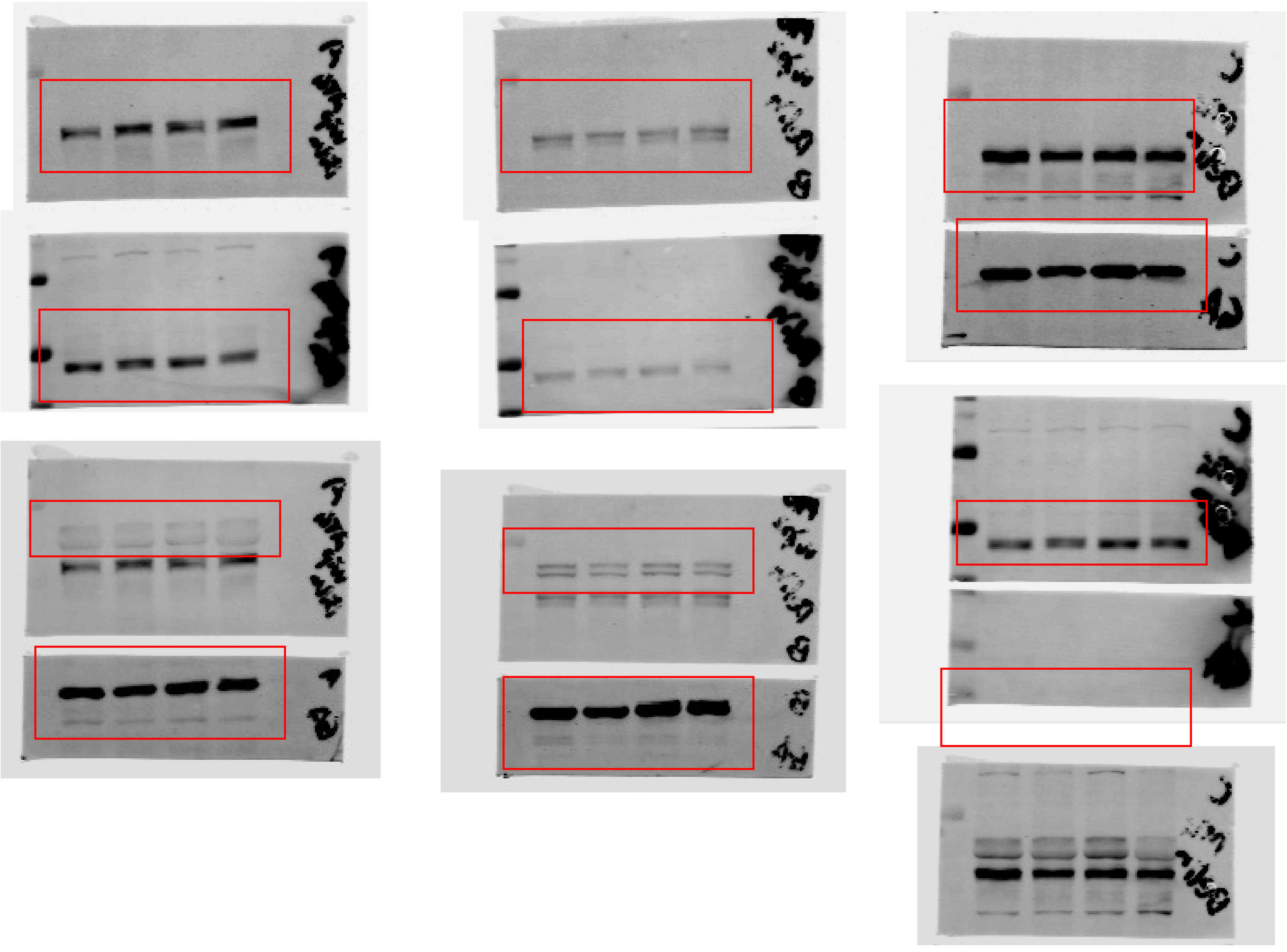

Fig S1E

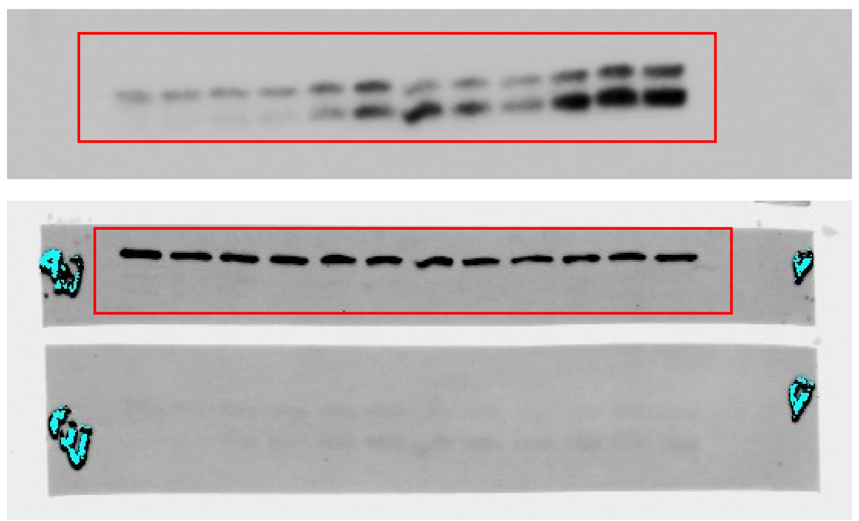

Fig S1F

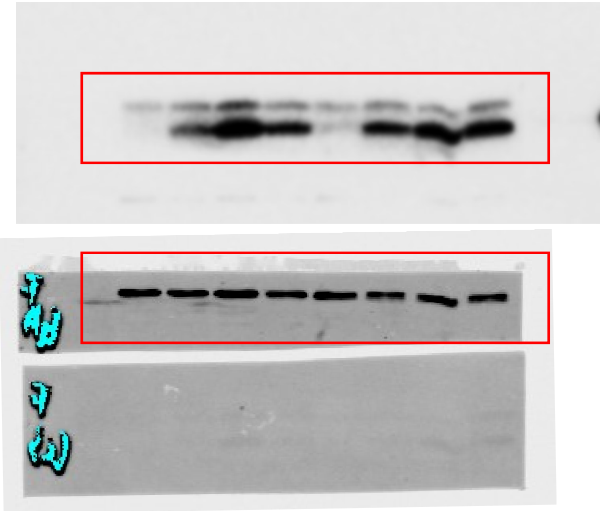

Fig S2C

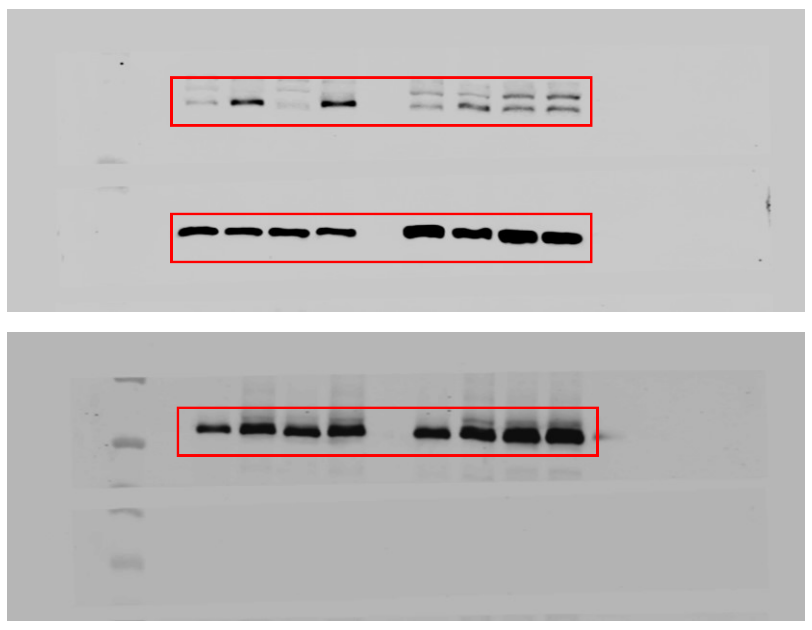

Fig S4A

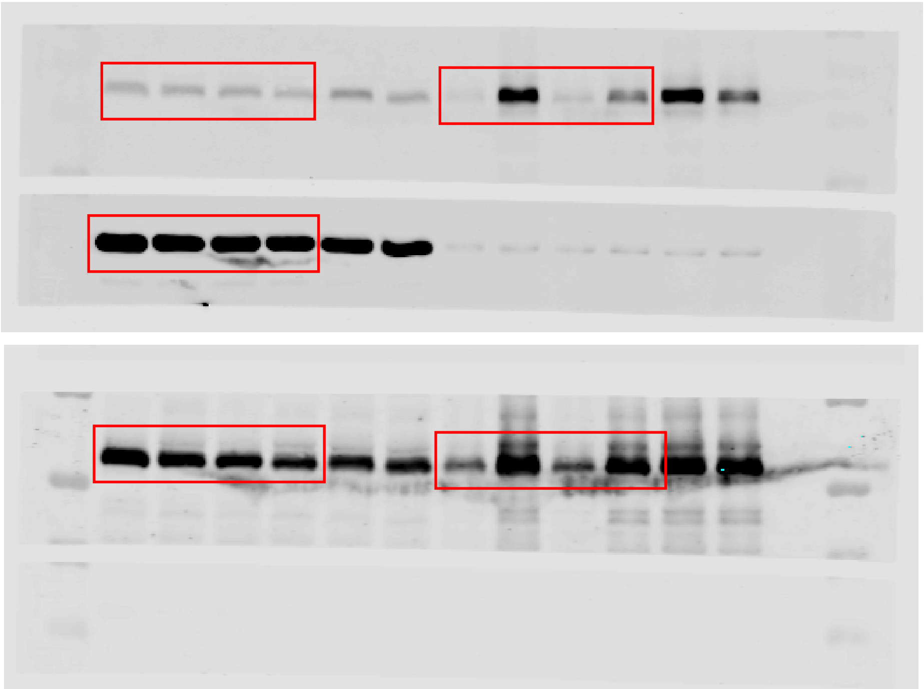

Fig S4B

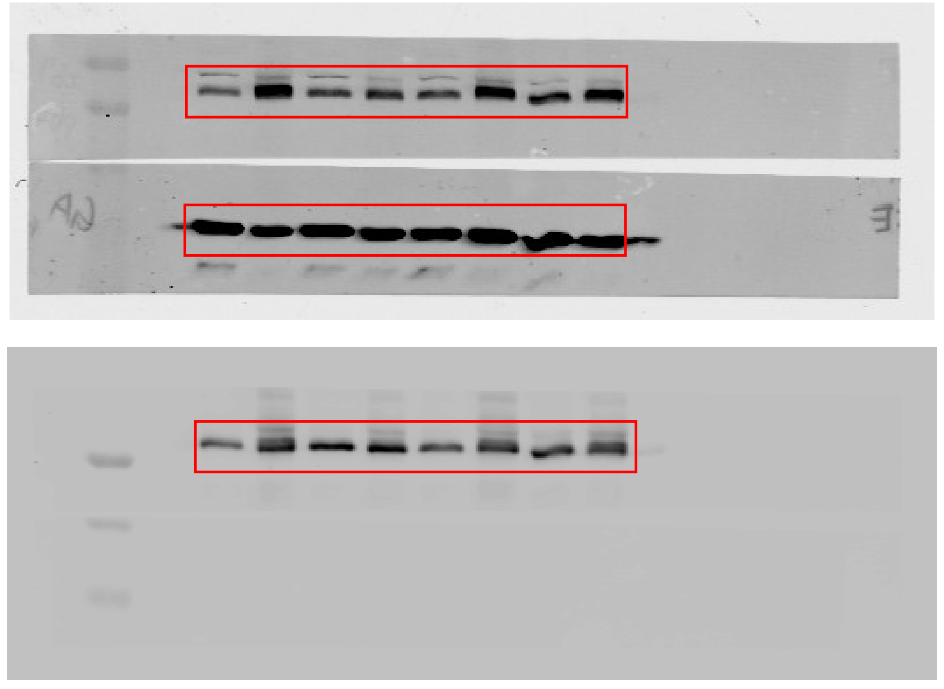

Fig S4D

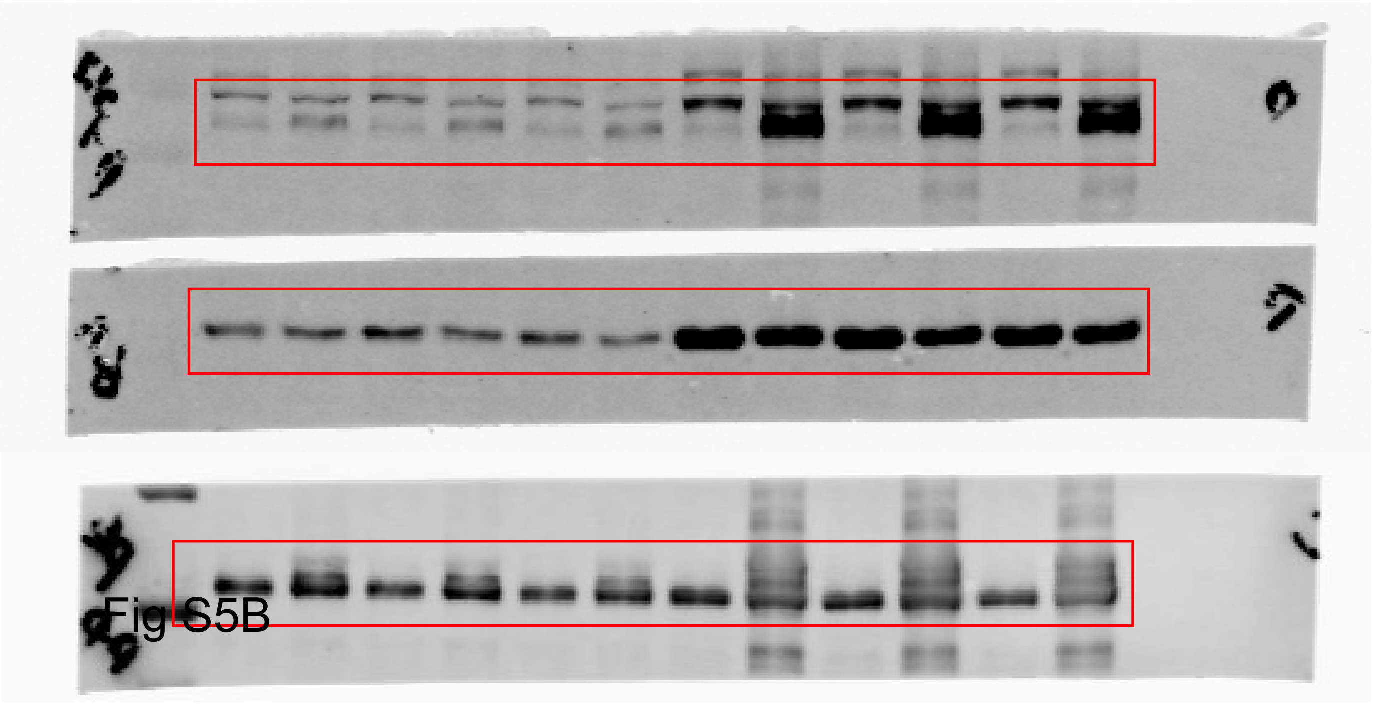

Fig S5A

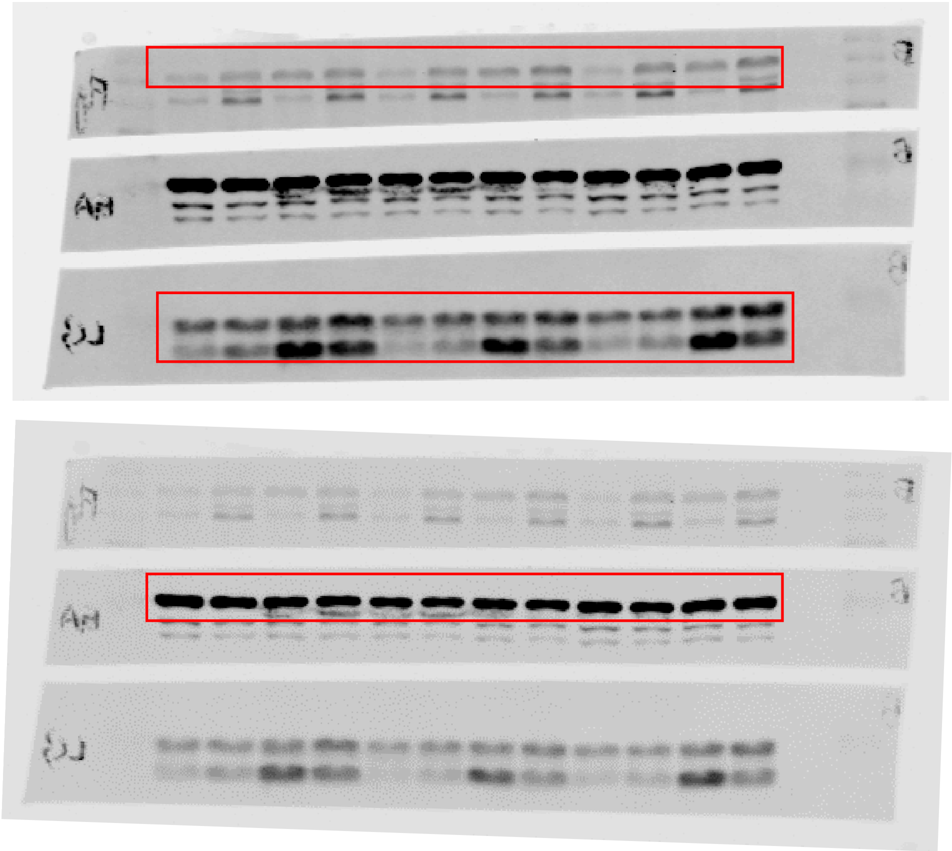

Fig S5B

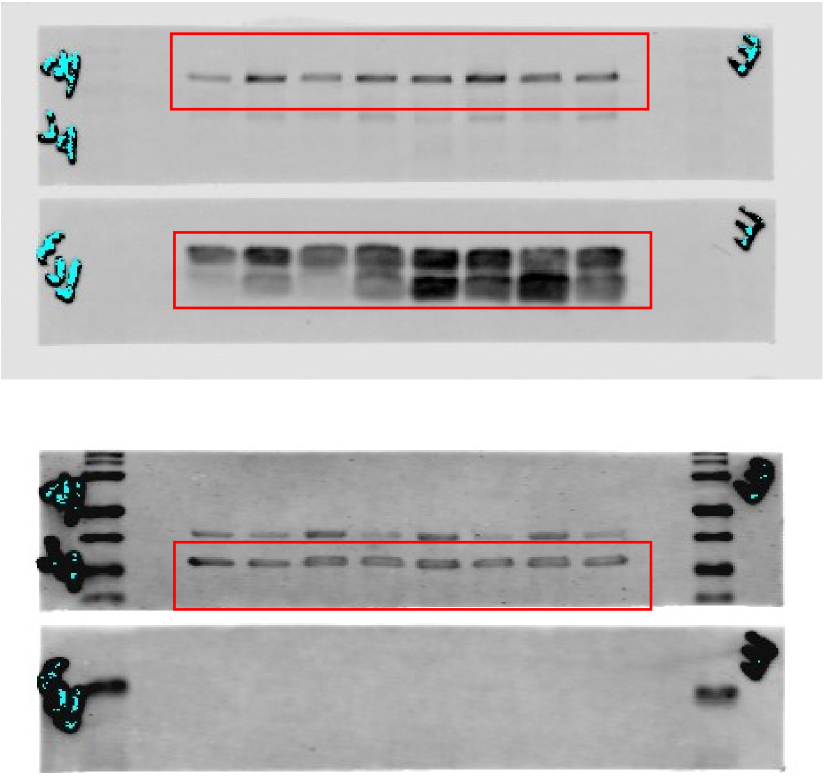

Fig S6A

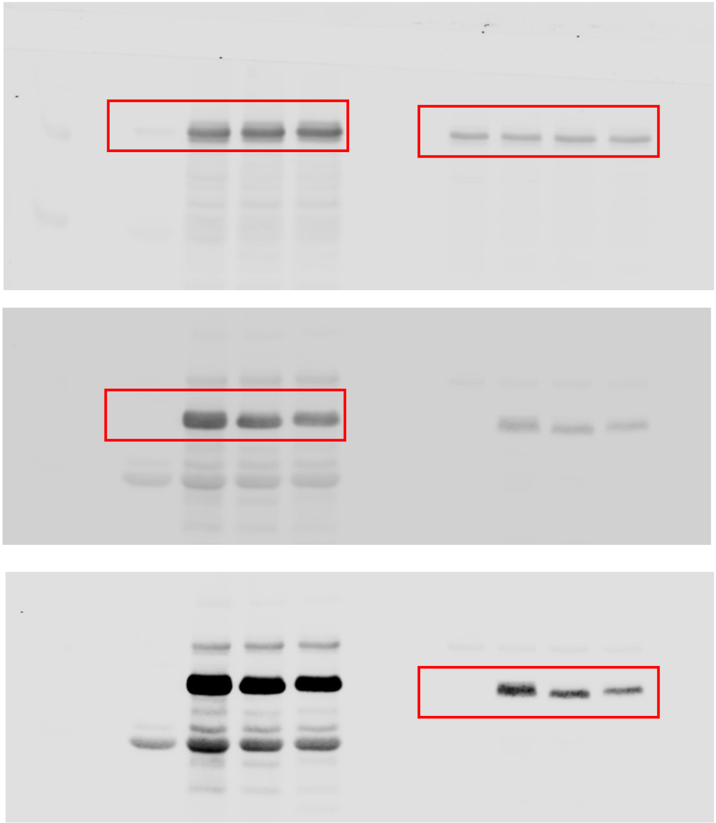

Fig S6B

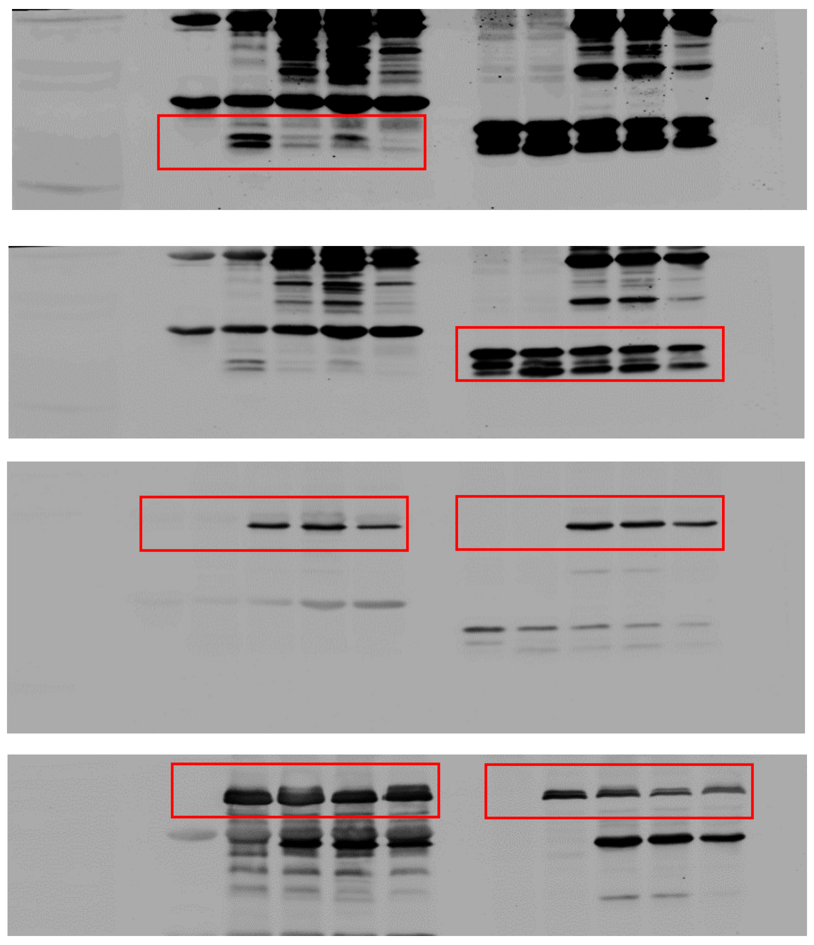

Supplement: Supplementary file 2 — Uncroupped WB [file 41419_2022_5061_MOESM2_ESM.pdf]
